# IPED: a highly efficient denoising tool for Illumina MiSeq Paired-end 16S rRNA gene amplicon sequencing data

**DOI:** 10.1186/s12859-016-1061-2

**Published:** 2016-04-29

**Authors:** Mohamed Mysara, Natalie Leys, Jeroen Raes, Pieter Monsieurs

**Affiliations:** Unit of Microbiology, Belgian Nuclear Research Centre (SCK-CEN), Mol, Belgium; Department of Bioscience Engineering, Vrije Universiteit Brussel, Brussels, Belgium; VIB Center for the Biology of Disease, VIB, Leuven, Belgium; Department of Microbiology and Immunology, REGA institute, KU Leuven, Belgium

**Keywords:** Error correction, Denoising, 16S rRNA gene amplicon sequencing, MiSeq, Metagenomics

## Abstract

**Background:**

The development of high-throughput sequencing technologies has revolutionized the field of microbial ecology via the sequencing of phylogenetic marker genes (e.g. 16S rRNA gene amplicon sequencing). Denoising, the removal of sequencing errors, is an important step in preprocessing amplicon sequencing data. The increasing popularity of the Illumina MiSeq platform for these applications requires the development of appropriate denoising methods.

**Results:**

The newly proposed denoising algorithm IPED includes a machine learning method which predicts potentially erroneous positions in sequencing reads based on a combination of quality metrics. Subsequently, this information is used to group those error-containing reads with correct reads, resulting in error-free consensus reads. This is achieved by masking potentially erroneous positions during this clustering step. Compared to the second best algorithm available, IPED detects double the amount of errors. Reducing the error rate had a positive effect on the clustering of reads in operational taxonomic units, with an almost perfect correspondence between the number of clusters and the theoretical number of species present in the mock communities.

**Conclusion:**

Our algorithm IPED is a powerful denoising tool for correcting sequencing errors in Illumina MiSeq 16S rRNA gene amplicon sequencing data. Apart from significantly reducing the error rate of the sequencing reads, it has also a beneficial effect on their clustering into operational taxonomic units. IPED is freely available at http://science.sckcen.be/en/Institutes/EHS/MCB/MIC/Bioinformatics/.

**Electronic supplementary material:**

The online version of this article (doi:10.1186/s12859-016-1061-2) contains supplementary material, which is available to authorized users.

## Background

The development of high-throughput sequencing technologies has revolutionized the field of microbial ecology by offering a cost-efficient method to assess microbial diversity at an unseen depth. Initial ecological applications mainly relied on the usage of the 454 pyrosequencing platforms, resulting in an impressive repository of bioinformatics analysis tools for processing this kind of data, as used for example in 16S rRNA gene amplicon sequencing data. Linking different tools developed for the preprocessing of amplicon sequencing data has resulted in frequently used analysis pipelines such as mothur [1], QIIME [2] and UPARSE [3].

Due to the recent advances in other high-throughput sequencing technologies regarding throughput and read length, and the announcement of Roche to shut down its 454 services by 2016, sequencing platforms provided for example by Pacific Biosciences and Illumina gain importance for assessing microbial diversity using amplicon sequencing. However, analysis pipelines developed for 454 pyrosequencing data cannot be translated into an Illumina MiSeq specific pipeline in a straightforward way due to fundamental differences between both sequencing technologies.

Indeed, the 454 pyrosequencing technology has difficulties in predicting the exact length of homopolymers, as such mainly leading to indel errors [4, 5]. Illumina sequencing data do not suffer from indel errors to the same extent, but rather from nucleotide substitutions (miscalling), mainly originating from two effects: 1) high correlation of the intensities of A and C as well as G and T due to similar emission spectra of the fluorophores [6–8], and 2) dependency of the signal of each cycle on the signal before and after this cycle, caused by inadequate flushing of fluorophores, incomplete removal of the 3’ terminators, or integration of nucleotides without effective 3’ terminators [6], known as phasing and pre-phasing. Additionally, it has been shown that such substitutions are often linked to the presence of the GGC motif, or more in general, to the GC-richness of the amplified region [9–11].

Different approaches have been developed for reducing sequencing errors originating from the Illumina MiSeq sequencing platform. These approaches can be categorized into three types: 1) denoising tools which actively resolve sequencing errors, 2) paired-end assemblers that merge overlapping reads into one contig represented by a consensus sequence (specifically for MiSeq amplicon paired-end sequencing data), and 3) quality filtering approaches which remove poor-quality reads or regions.

A large number of denoising algorithms has already been developed for 454 pyrosequencing reads, for example Denoiser [12], AmpliconNoise [13], SLP [14], Acacia [15] and NoDe [16]. However, due to fundamental differences in the nature of both sequencing technologies, denoising algorithms developed for 454 pyrosequencing data are likely to perform suboptimal when applied to Illumina sequencing data in a naive way. At this moment, a few algorithms for denoising Illumina MiSeq paired-end amplicon sequencing data have been developed. One of the 454 pyrosequencing denoising algorithms, i.e. SLP [14] (implemented as pre.cluster in mothur) and the more recently released algorithm called UNOISE [17], have been shown to be applicable to Illumina MiSeq specific analysis pipelines [5].

However, next to denoising tools, a plethora of tools has been developed for assembling paired-end reads into one amplicon contig, by merging both the forward and reverse reads into one consensus sequence. Apart from the assembly tools integrated in the amplicon sequencing pipelines like mothur, QIIME and USEARCH, other more general paired-end assembly algorithms have been developed such as FLASH [18] PANDAseq [19], COPE [20] and PEAR [21]. Additionally, several quality filtering approaches were implemented to identify and remove or trim reads with poor quality, according to specific criteria defined within each tool, as implemented in mothur, QIIME and USEARCH. Despite the fact that paired-end assemblers and quality filtering approaches cannot be seen as genuine denoising tools, they will have an effect on the error rate, and should be included in a benchmark when assessing denoising tools.

In this work we propose the Illumina Paired-End Denoiser (IPED) algorithm, an error correction algorithm specifically developed for denoising Illumina MiSeq 16S rRNA gene amplicon sequencing data. Our machine learning methodology was benchmarked using four different mock datasets, each of them containing sequencing data of different hypervariable regions in the 16S rRNA gene, including completely as well as partially overlapping paired-end reads. Working with mock communities consisting of a known set of species had the major benefit that we can use the error rates as most prominent evaluation criterion. Since the amount of data produced within one sequencing run is steadily increasing for the Illumina MiSeq technology, we evaluated the additional computational cost associated with our algorithm.

## Methods

### Mock communities

Four publicly available Illumina MiSeq sequencing datasets of mock communities were used within this work. The first mock community – called MOCK1 – is composed of 21 species added in equimolar concentrations (5 ng/μl) [5]. The second mock community, termed MOCK2, has almost the same composition as MOCK1, however omitting one species, resulting in 20 different organisms [22]. The DNA of the mock communities can be obtained from BEI Resources (catalog number HM-278D). Both Illumina MiSeq libraries were prepared using primers as described in the work of Kozich et al. [5] for the amplification of the V34, V4 and V45 hyper-variable regions of the 16S rRNA gene for MOCK1, and in the work of Nelson et al. [22] for the amplification of the V4 and V45 region for MOCK2. Both mock communities were sequenced on the Illumina MiSeq platform using the 2 × 250 bp paired-end protocol. Merging both reads into one contig resulted in different contig lengths for each primer pair: contigs resulting from the V4 primer pairs in MOCK1 and MOCK2 resulted in a length of 251–253 bp (completely overlapping paired-end reads), V45 contigs in MOCK1 and MOCK2 in lengths of 375 nt and 390 nt respectively (overlapping regions of 125 and 110 bp respectively) and V34 contigs in MOCK1 in a length of 430 nt (overlapping region of 70 bp). In MOCK1, four sequencing runs were performed with various cluster densities (ID's: 130401,130403, 130417 and 130422). In MOCK2, samples for each region (V4 and V45) were run in duplicate (named v4.I.1, v4.I.05, v4.v5.I.1 and v4.v5.I.11 respectively).

The third mock community – called MOCK3 – consists of three samples (named M1, M2, M3), each consisting of 12 species (Sequencing Read Archive accession number SRP066114). The MOCK3 community was sequenced on the Illumina MiSeq platform using the 2 × 300 bp paired-end protocol. Merging both reads into one contig resulted in a length of 422–428 bp after clipping the primers (overlapping regions of 141 bp). Table 1 provides a detailed description of the MOCK1, MOCK2 and MOCK3 sequencing datasets.

**Table 1:** Overview of the mock sequencing data discussed in this work. It contains information on the amplified regions, samples ID’s, number of paired-end reads (i.e. contigs), average contig length (i.e. length after merging both paired-end reads), and average length of the overlapping part between both paired-end reads

Name	Region	length	Overlap	ID	#contigs
MOCK1 [5]	V34	430	70	130401	184216
				130403	131241
				130417	102547
	V4	250	250	130422	79701
				130401	1217529
				130403	1191998
	V45	375	125	130417	1015673
				130422	871118
				130401	826262
MOCK2 [22]	V4	250	250	v4.I.1	213043
				v4.I.05	240682
	V45	390	110	v4.v5.I.1	2484
				v4.v5.I.11	90126
MOCK3 (SRP066114)	V34	421	140	M1	35168
				M2	60488
				M3	21723

The fourth mock community - called MOCK4 - consists of 73 samples and was recently published in Schirmer et al. [11]. Their microbial composition ranges from a single species to diverse mock communities (49 bacteria and 10 Archaea), mixed either in even or uneven concentrations. Five different Illumina MiSeq library preparation methods were used to amplify the V4 and the V34 region. Contigs constructed by merging both reads resulted in two different lengths, ranging from 253 nucleotides (i.e. almost completely overlapping reads) to 450 nucleotides (partially overlapping reads). A detailed description of MOCK4 and the sequencing protocols used can be found in the original publication [11].

### Pre-processing steps

For all datasets (MOCK1, MOCK2, MOCK3 and MOCK4) contigs were created by merging the paired-end reads using a heuristic based on the difference in Phred quality scores of both reads as implemented via the make.contigs command in mothur [5]. Contigs were culled if they had an ambiguous base or if they were not properly merged. All sequencing data were trimmed, aligned, screened, filtered and dereplicated using the mothur software package (v.1.33.3), thereby following the SOP as described on the mothur website (http://www.mothur.org/wiki/MiSeq_SOP d.d. 2015-05-18). Afterwards, reads were denoised (by any of the tested denoising algorithms) and chimeras were identified using the “seq.error” command in mothur [1] using the full length 16S rRNA genes of the mock community species as reference. This “seq.error” command was also used to identify sequencing errors. To assess the performance of our newly introduced algorithm, IPED was benchmarked against the Pre-cluster and UNOISE algorithms, both using the recommended parameter settings as proposed by the initial developers (described in the mothur MiSeq SOP and the UNOISE publication respectively [17]).

Despite the fact that paired-end assembly algorithms cannot be considered as denoising algorithms in the strict sense, their potential influence on the error rate required a comparison with the error rates obtained using IPED. Therefore, different paired-end assembly algorithms used for amplicon sequencing were tested on the MOCK1, MOCK2 and MOCK3 datasets. Algorithms included are the standalone tool PEAR as well as the assembly steps as included in mothur, QIIME and USEARCH, together with their proposed quality filtering. An overview of the commands used for those tools is given in Additional file 1: section 4.

For MOCK1, MOCK2 and MOCK3 the sequencing reads were clustered in operational taxonomic units (OTU) using the mothur recommended clustering approach (shown in Additional file 1: section 8) as well as UPARSE [3] with default parameters, with the exception of singleton removal. The default setting of singleton removal was deactivated to accurately assess the effect of sequencing errors on all the OTUs produced, including singletons. The exact commands are given in Additional file 1: section 3.

### Training data

An important component of the IPED algorithm is the machine learning method developed to predict potentially erroneous positions. A dedicated dataset for training and testing this machine learning method was created by randomly selecting reads from sample 130401 of MOCK1 (1,000 reads from the V34, V4 and V45 region respectively), resulting in a dataset of 3,000 reads. Important to notice is that all three samples used to construct the training data were completely disregarded in the subsequent benchmarking analysis. Each nucleotide in those reads was evaluated as being either erroneous (mismatch, insertion or deletion) or correct, identified as such by aligning those reads against the reference genomes. In order to obtain highly reliable training instances, the same parameter setting was applied as used in Gilles et al [23] for running BLAST [24] and subsequently ClustalW [25]. This led to a dataset consisting of 1,031,625 instances (i.e. all nucleotide positions in the 3,000 reads). These data were cleaned as follows: dereplication, randomization and simplification via selecting a subset of the features (see further for details on the feature selection step). Next, the data were split into three subsets, thereby respecting the initial ratio between erroneous versus non-erroneous instances throughout the three subsets: (a) a learning data set for training the classifier, (b) a validation set for selecting the most optimal kernel and (c) a test data set for testing the accuracy of the classifier. Subset (a) and (b) were further modified by adjusting the ratio between erroneous and non-erroneous instances: for subset (a), several ratios between erroneous and non-erroneous instances were applied to select the one resulting in the best performance when training the classifier, while for subset (b) we used an equal ratio between both classes. Extra information on the feature-selection step and selecting the correct ratio in subset (a) is given in section 1 of Additional file 1. All machine learning methods for training and testing IPED were used as implemented in the WEKA software version 3.7.11 [26].

### Evaluation parameters

For the evaluation of IPED, we calculated the number of true positives (TP), false negatives (FN), true negatives (TN) and false positives (FP) as follows: if an erroneous nucleotide was correctly detected as such, it is a TP, if it was not it is a FN, if an non-erroneous nucleotide was correctly detected as such it is a TN, if not it is a FP. We used the Mathews Correlation Coefficient (MCC) [27]: (TP × TN-FP × FN)/√((TP + FP)(TP + FN)(TN + FP)(TN + FN)), sensitivity (i.e. the proportion of actual erroneous positions that was detected as such: TP/(TP + FN)), specificity (i.e. the proportion of actual non-erroneous positions that was detected as such: TN/(TN + FP)), and Receiver operating characteristics (ROC). The latter analysis combines both sensitivity and specificity by plotting the sensitivity (Y axis) against one minus the specificity (X axis)). ROC curves were produced by swiping the threshold cut-off of the probability estimated by each classifier, and plotting the sensitivity versus one minus the specificity value.

## Results

Our newly developed algorithm for denoising Illumina MiSeq amplicon sequencing data was developed in two steps. First an artificial intelligence classifier was trained to detect potentially erroneous positions in the sequencing reads. Secondly, a modified version of the previously published algorithm (Pre-cluster) [28] (which is the mothur implementation of the Single Linkage Preclustering algorithm [14]), was adapted in such a way that it does not penalize those potentially erroneous positions during clustering. The development of both the classifier and clustering component of the IPED algorithm is discussed in the first part of this results section. Once the setup and training of IPED has been finalized, the algorithm was tested on a wide range of datasets against Pre-cluster and UNOISE, and this at the level of error rate, computational cost and the accuracy of the OTU clustering.

### IPED development

#### IPED classifier

The performance of the IPED algorithm is largely depending on the ability of its classifier component to correctly identify erroneous positions. The machine learning approach followed to develop this classifier is based on supervised learning, where we trained the classifier to identify such positions by training it on a dedicated data set (i.e. a data set containing correct and erroneous positions). Training of the IPED classifier consisted of four consecutive stages: 1) identify those parameters that are potential predictors of sequencing errors (i.e. feature identification), 2) select the most informative parameters (i.e. feature selection), 3) train the classifier to identify sequencing errors based on those parameters (i.e. model training), and 4) check whether the classifier correctly predicts sequencing errors on unseen sequencing data (i.e. validation).

The first stage consisted of extracting a list of features potentially predicting erroneous positions. Different sequencing characteristics have been taken into consideration such as the position in the read, the homopolymer status and Phred quality score, the presence of the GGC motif in front of the position in question, the homopolymer status (“0” in case of no homopolymer, “-1” when the nucleotide is ambiguous, and in case of a homopolymer an ascending number indicating the position within the homopolymer) and the Phred quality score of the preceding and succeeding position for both the forward and reverse reads, totaling up to 16 features. An additional feature was added indicating whether the nucleotide was situated in the overlapping region of both paired-end reads, and if so to indicate whether they have no conflict, a conflicting base call (mismatch, deletion or insertion), or unknown overlap (if at least one of both nucleotides is ambiguous).

Importantly, integrating too many uninformative features would have led to an inflation of the computational cost. Accordingly, reducing the total number of available features (17 in total) while retaining the predictive power of our algorithm has a beneficial effect on the performance of the classifier as it increases its accuracy and reduces the computational cost. Therefore, in a second stage, a feature-selection step was applied using a three-fold cross-validation on the training data. This approach allowed us to identify those features having a high predictive power for recognizing erroneous positions (i.e. having a high correlation with the class ‘error’ or ‘non-error’), while having a low correlation with other predictive features. Performing this step resulted in a subset of six features i.e. for the forward read: the position in the read, homopolymer status and Phred quality score; for the reverse read: the position in the read and Phred quality score; and as last feature the overlap status between the forward and reverse read. Important to notice within this context is the absence of the GGC motif after the feature selection step. However, this might not be surprising as we could clearly see a drop in the Phred quality score in the positions succeeding this motif (data not shown). As the Phred quality score was retained after applying the feature-selection step, the GGC motif was removed from the features list. This could be explained either by the weak evidence of error incidences related to the presence of the GGC motif [29] or due to the high correlation between this motif and the Phred quality score, making this feature superfluous.

In the third stage, these six features were used to train a wide range of classifiers available in WEKA based on subset (a) of the training data. The goal of this step was to select the type of classifier that obtains the highest accuracy in predicting erroneous positions. In order to optimize the balance between specificity and sensitivity, a range of ratios balancing the number of erroneous versus non-erroneous instances was tested. The highest sensitivity while maintaining an acceptable specificity was obtained using a ratio of 1:3 (error : non-error) (an overview of different ratios is available within Additional file 1: section 1). The training process was further evaluated by plotting the learning curves for each of the classifiers, which confirmed that a training dataset size consisting of 5,000 erroneous and 15,000 non-erroneous instances (respecting the 1:3 ratio) gave the best performance (see Additional file 1: section 2). Subset (b) of the training data was used to evaluate each of the individual classifiers using sensitivity, specificity, MCC measurements and ROC analysis as performance parameters. Additional analyses tested the performance obtained when different sets of classifiers (ranging from two up to five classifiers) were combined using plurality voting. In a plurality voting approach, each of the considered classifiers outputs a confidence score for the classification made and the class with the highest confidence is selected as output for the respective instance. Comparing the performance of the individual classifiers as well as the different voting combinations (see Additional file 1: section 2), the best performance was achieved via plurality voting combining Multilayer Perceptron (MLP – with a learning rate of 0.3, momentum of 0.2, and using 6 perceptrons in one hidden layer) and Random Forest (using an ensemble of unpruned decision trees). This combination achieved a sensitivity of 0.57, specificity of 0.93, MCC of 0.53 and ROC area under the curve (AUC) of 0.87 on subset (b) of the training data, thereby outcompeting the other machine learning approaches. The additional computational burden resulting from this plurality voting approach was minimal, as the best performing single classifier (random forest) required 2.2 sec compared to 2.3 sec for the plurality voting approach (including MLP and random forest) (tested on subset (c) of the training data, containing around 200,000 instances).

#### IPED clustering

**Fig. 1:**
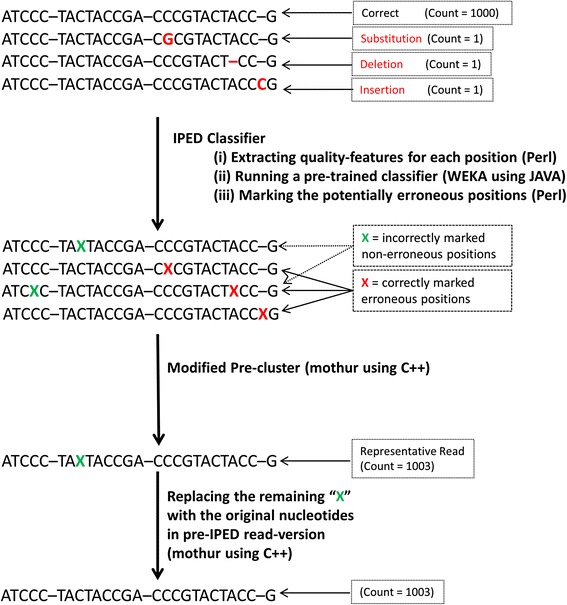
Schematic overview showing the different steps of the iped algorithm

As mentioned above, the first step in the development of IPED consisted of training a classifier able to predict potentially erroneous positions with high accuracy. In the second step a modified version of the SLP algorithm [14] as implemented via the Pre-cluster command in mothur [28] has been developed [16]. In the original Pre-cluster implementation, sequences are sorted based on their abundance level in descending order. When a rare sequence is at maximum 1 nt per 100 nt differing from a more abundant one, it is merged with the more abundant one and its abundance is added to the latter one. In the first step of IPED, the classifier has marked some of the positions as potentially erroneous. We have developed a modified version of the mothur Pre-cluster algorithm that will not penalize those marked positions when calculating the amount of conflicting positions between two reads. This means that any position in the alignment containing a nucleotide which is marked as potentially erroneous, will not increase the distance score (i.e. the score used as cut-off to either merge two reads, or leave them ungrouped). After the clustering step, those masked positions are reverted to their original nucleotide as they were before running IPED. A schematic representation of this approach is given in Fig. 1. The IPED software can be downloaded via https://github.com/M-Mysara/IPED or http://science.sckcen.be/en/Institutes/EHS/MCB/MIC/Bioinformatics/.

### Benchmarking of IPED

#### Impact of denoising algorithms on the error rate

Once the development of the IPED algorithm has been finalized, its performance was compared with those of the Pre-cluster and UNOISE algorithms. Both those tools are the only denoising algorithms currently applicable for Illumina amplicon sequencing data. Using the reference 16S rRNA gene sequences from the organisms present in the MOCK1, MOCK2 and MOCK3 communities, we calculated the error rates before applying the denoising algorithms, which were subsequently compared with the error rates obtained after applying IPED, Pre-cluster and UNOISE on all three mock datasets. The error rate was calculated using the seq.error command by taking the ratio of the number of deletions, insertions and substitutions over the total number of bases.

The average error rate before denoising (i.e. after using the make.contigs command in mothur) was 0.0005 and 0.0006 for V4 (MOCK1 and MOCK2 respectively), 0.0071 and 0.0050 for V45 (MOCK1 and MOCK2 respectively) 0.0026 and 0.0015 for V34 (MOCK1 and MOCK3 respectively). The fact that the error rate of V4 was up to an order of magnitude lower than both other regions is not surprising since the V4 amplicon fragment consists of two completely overlapping reads, as such assuring a two-fold prediction for each nucleotide.

When comparing the output of IPED with the raw error rates, our algorithm was able to reduce on average the error rate with 72 % (individual values for different regions and runs are varying between 28 % and 94 %). When benchmarking those results with UNOISE and Pre-cluster, UNOISE was able to reduce the error rate on average by 52 % (individual values varying between an increase of the error rate with 66 % and a decrease of 97 %) while Pre-cluster was able to reduce it by 51 % (individual values varying from 4 % to 86 %) (see Table 2 for all details). On average (averaged over all mock communities), IPED diminished the error rate to 0.0010, while UNOISE and Pre-cluster reduced the overall error rate to the same value of 0.0018 (see Table 2). However, compared to other denoising algorithms, the effect of IPED is more pronounced for those regions with no complete overlap between both paired-end reads (i.e. region V34 and V45). Importantly, it should be noted that UNOISE (as implemented in USEARCH) results in an additional loss of on average 13 % of sequencing data due to its more stringent pre-processing steps, as illustrated in supplementary file 1 section 4.

**Table 2:** Overview table comparing error rates of the samples treated with UNOISE (after USEARCH preprocessing) and those without applying a denoising algorithm, after applying Pre-cluster or after applying IPED (after mothur preprocessing). Due to the difference preprocessing steps applied in USEARCH and mothur, the amount of reads removed differ, where around 53 % and 39 % of reads are removed in respective order

Variable region	Sample ID			Error rates	
		USEARCH + UNOISE	Mothur (make.contigs)	Mothur + Pre-cluster	Mothur + IPED
V34	130403	0.0003	0.0026	0.0013	0.0002
	130417	0.0004	0.0023	0.0010	0.0003
	130422	0.0008	0.0028	0.0017	0.0008
	M1	0.0006	0.00149	0.0007	0.0004
	M2	0.0007	0.00150	0.0008	0.0006
	M3	0.0005	0.00140	0.0007	0.0005
V4	130403	0.00011	0.00056	0.00013	0.00010
	130417	0.00009	0.00051	0.00010	0.00008
	130422	0.00009	0.00049	0.00010	0.00008
	v4.I.1	0.00002	0.00061	0.00008	0.00004
	v4.I.05	0.00002	0.00068	0.00010	0.00004
V45	130403	0.0030	0.0084	0.0055	0.0022
	130417	0.0029	0.0069	0.0041	0.0020
	130422	0.0026	0.0060	0.0033	0.0016
	v4.v5.I.1	0.0082	0.0066	0.0061	0.0041
	v4.v5.I.11	0.0084	0.0033	0.0034	0.0031
Average	All samples	0.0018	0.0029	0.0018	0.0010

The same trend in lowering the error rate was observed when running IPED on the MOCK4 dataset. Indeed, when both reads are almost completely overlapping (contig lengths ranging between 253 and 292), IPED was able to reduce the error rate from 0.0041 to 0.0032. This effect was more prominent when dealing with contigs with a smaller overlap between both paired-end reads (contig length ranging between 330 and 450), showing a decrease in the error rate from 0.0065 to 0.0033 (see Additional file 1: section 5). However, important to mention within this context is the elevated error rate of this data set, which is significantly higher than should be expected for Illumina MiSeq sequencing data. As such, caution should be given when extrapolating those results.

**Fig. 2:**
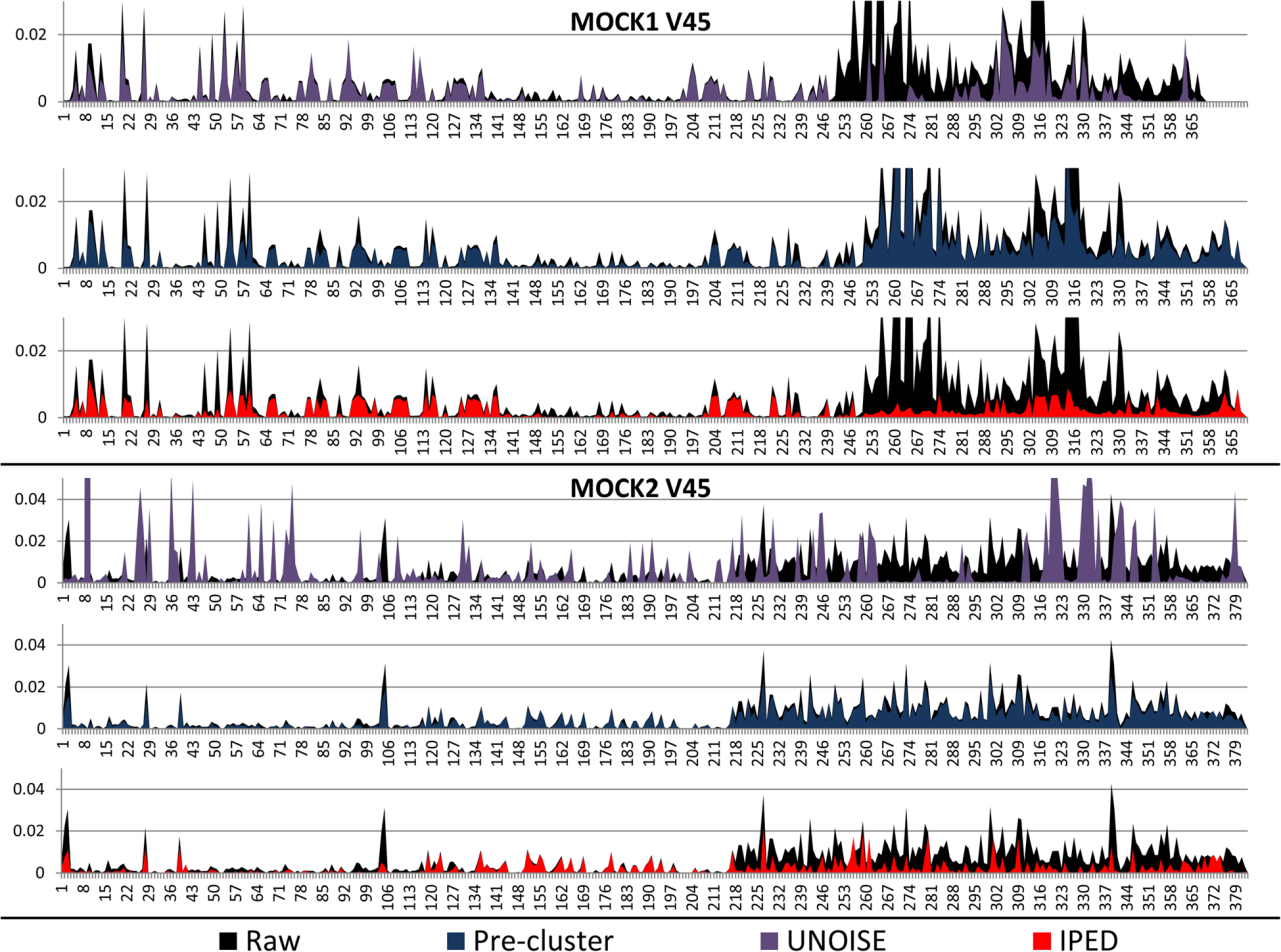
Plot showing the error rate versus the position in the read after being treated with Pre-cluster (*blue*), UNOISE (*violet*) and IPED (*red*). The raw error rates (i.e. without applying a denoising algorithm) are colored *black*

Plotting the error rates for the MOCK1 and MOCK2 datasets versus their position in the amplicon, indicated that the beneficial effect of IPED is mainly situated in the uniquely covered region of the second read (i.e. those positions not overlapping with the first read), and to a lesser extent also the overlapping part (i.e. those positions sequenced twice: a first time via read one, and a second time via read two) (see Fig. 2 for the V34 and V45 region of MOCK1, and section 6 in Additional file 1 for more details).

As stated in the introduction, a plethora of algorithms is available for assembling paired-end reads into one amplicon fragment. Even though those algorithms are not denoising algorithms in the strict sense, they can have an impact on the error rate of the resulting fragment. For this comparison, error rates were calculated for MOCK1, MOCK2 and MOCK3 after running different assembly algorithms, being fastq_mergepairs (USEARCH), make.contigs (mothur), join_paired_ends (QIIME) and PEAR, which resulted in error rates of 0.0027, 0.0029, 0.0031 and 0.0097 respectively (see Additional file 1: section 4). Important to notice is that the number of reads retained is correlated with the error rate: PEAR returned the highest error rate, however it managed to retain 86 % of the reads, while USEARCH reached the lowest error rate but removed more than 53 % of the data. Anyhow, it is clear from those data that the error rates obtained using those assembly algorithms did not come close to the error rates obtained with IPED (run on the output of the mothur make.contigs step) i.e. 0.0010. Similar effects were obtained by including IPED after the QIIME assembly step, leading to an error rate of 0.0014, which was also significantly lower than the error rate obtained with the assembly steps solely (data not shown). Those data suggest that whatever currently available assembly algorithm is used, running IPED afterwards will still have a beneficial effect on the error rate.

To investigate the extra computational cost related to IPED, the calculation time was registered for all three samples of the MOCK1 dataset covering the V4 region, where each sample was subsampled to 6000 unique reads. When one processor (single Intel Xeon E5-2640 2.50 GHz CPU) was used for each sample (i.e. a total of three processors), IPED required 70 s for running all three samples, while Pre-cluster and UNOISE could end the analysis in 14 s and 12 s respectively. Similar relative differences in calculation time were also observed for other MOCK1, MOCK2 and MOCK3 samples (see Additional file 1: section 7).

#### Impact of denoising algorithms on the OTU clustering

As the negative effect of sequencing errors has an influence on the amount of spurious OTUs, ideally an improvement at the level of denoising step should be reflected in a decrease of the number of OTUs. Although the OTU clustering step is influenced by the number of reads and level of complexity in the mock samples, [5], it has been used by others as a metric for sequence quality [5, 12–14, 16, 17, 28, 30]. In order to get an idea to which extent IPED, UNOISE and Pre-cluster have a beneficial effect on the OTU clustering step, sequencing data denoised by either one of the three approaches were clustered using the average neighborhood hierarchical clustering algorithm via the “cluster” command (as implemented in mothur), and subsequently compared with the number of OTUs obtained when no denoising algorithm was applied. As the amount of species present in both mock communities is known, in the most ideal scenario the amount of OTUs returned should be 20, 19 and 12 for MOCK1, MOCK2 and MOCK3 respectively. It is important to emphasize that any undetected chimera or possible contamination would lead to an inflation of the number of the OTUs. However, as their effect is the same for all tools, we assume that the number of OTUs still provides a good indication of the performance.

The average number of OTUs produced when the denoising step was omitted returned on average 109, 64 and 127 OTUs for the V4, V34 and V45 regions respectively (combined results of MOCK1 and MOCK2, except for the V45 region of MOCK1 – see further). IPED was able to reduce these numbers to an average of 94, 48 and 81 while Pre-cluster resulted in 110, 66 and 118 OTUs respectively and UNOISE resulted in 120, 15 and 363 OTUs respectively. Again, it is important to highlight the impact of the strict pre-processing approach followed by UNOISE, resulting in a removal of almost all of the reads in MOCK1 V34 samples in this pipeline, and therefore returning a very low number of OTUs for the UNOISE approach. Similarly, for MOCK3, the number of OTUs for the non-denoised data was 90, while integrating IPED, UNOISE or Pre-cluster in the preprocessing pipelines led to 84, 89 and 107 OTUs respectively. Altogether, this analysis showed a more beneficial effect of IPED on the OTU clustering step than Pre-cluster and UNOISE (see Additional file 1: section 8.1). Concerning the number of OTUs for the V45 region of MOCK1, it was not possible to calculate the number of OTUs due to high memory requirements, leading to the exclusion of these data sets from the OTU analysis.

It should be noted that all mock samples analysed in this work contain a high number of reads per sample (on average more than 500,000), which is significantly higher than the number of sequences obtained for most real-life microbial diversity studies. In order to work with more realistic numbers, we rarified the datasets to 5,000 – as proposed in Kozich et al. [5] – and 25,000 reads per sample. Again, IPED outperformed UNOISE and Pre-cluster when applied on the rarefied MOCK1, MOCK2 and MOCK3 samples (see Additional file 1: section 8). Moreover, similar results were obtained upon using the UPARSE clustering algorithm on both complete and rarefied datasets (see Additional file 1: section 8.1).

However, the analysis performed above starts from an ideal situation, since all chimeras can accurately be removed using the reference sequences from the species present within the mock community. Additionally, the species present in the mock communities are well-known species, incorporated in the reference alignment database, as such resulting in an accurate alignment. To get an idea on the effect of the different denoising tools in case of a more realistic scenario, i.e. using a regular chimera removal algorithm and the presence of species not represented in the reference alignment database, we applied a regular chimera detection tool (CATCh de novo) [31] on the MOCK3 dataset in order to remove chimeric sequences from the mock community. Additionally we removed the corresponding sequences of the species represented in the mock community from the 16S rRNA reference alignment database, together with any other sequence showing a similarity higher than 97 % to any of those twelve species. As reported in section 11 of the Additional file 1, IPED was able to outperform Pre-cluster and UNOISE with a reduction of the error rate in the same range as reported earlier, and additionally led to the lowest number of OTUs.

In order to check the effect of the denoising algorithm on the OTU clustering quality, sequencing data were analysed using a preprocessing pipeline where the only varying factor was the denoising algorithm (IPED, Pre-cluster or UNOISE). This way we could assess whether the anticipated species of the mock community could be retrieved. Applying Pre-cluster and UNOISE led to less accurate clustering results as reads originating from the same species where more frequently scattered over different OTUs. In general we can conclude that applying IPED has a beneficial effect on the OTU clustering step when compared with the Pre-cluster and UNOISE results, since for all mock samples the number of OTUs produced with IPED was the closest to the actual number of species. Details on the number of OTUs are given in section 8 of the Additional file 1.

As a proof of principle, IPED was applied on a real data set (i.e. a non-mock dataset) to emphasize the effect on a more complex dataset. However, it is important to stress that unfortunately for those real-life datasets no error rates could be calculated, forcing us to revert to the number of OTUs as evaluation criterion. Despite the fact that this criterion is inferior to the error rate, it has been used in previous publications [30, 32, 33]. In this data set, presented in Kozich et al. [5], murine fecal samples of mice were used to assess the shifts of the microbial community after weaning at two different stages: early (0–9 days) versus late (141–150 days) after weaning. IPED was able to reduce the number of spurious OTUs, as illustrated by the rarefaction curves, and produced a more clear separation of clusters of late versus early stage samples when visualized in principle coordinate analysis (PCoA) biplots (see Additional file 1: section 9).

## Discussion

New sequencing technologies have revolutionized the assessment of microbial diversity via amplicon sequencing. However, each of the currently available high-throughput sequencing platforms suffers from sequencing errors originating from the sequencing technology itself (which is different from PCR point errors). In order to prevent the inflation of artificial OTUs due to these sequencing errors, different algorithms have already been developed for the correction of sequencing errors in 454 pyrosequencing data, for example SLP [14], AmpliconNoise [13] and Denoiser [12]. However, assessment of bacterial diversity using the Illumina MiSeq technology is now the standard, as it offers high throughput in combination with an acceptable read length. Recently, the SLP-based algorithm Pre-cluster (available as pre.cluster in mothur) and UNOISE (available in USEARCH) have been proposed as denoising tools in Illumina MiSeq specific pipelines [5, 17]. In this work, we introduced IPED as the best denoising tool specifically oriented towards Illumina MiSeq 16S rRNA paired-end reads.

IPED was shown to outperform Pre-cluster and UNOISE, as observed on the three mock datasets where our newly introduced algorithm could on average correct double the amount of sequencing errors. This effect seems to be less pronounced in those paired-end reads having a complete overlap between both reads, as every nucleotide position in the amplicon is covered twice, once by the first read, and once by the second read. Therefore, the added effect of IPED is smaller in those latter cases.

Moreover, reducing the error rate has a significant effect on the quality of the reads in the OTUs. Adding an error-correction step before running the OTU clustering algorithm, led to a very close correspondence between the number of OTUs returned, and the true number of species known to be present in the mock communities. Such a significant correspondence could not be obtained when omitting the denoising algorithm in the amplicon sequencing preprocessing pipeline, or via using any other denoising tool. However, caution should be given when extrapolating this to real-life environmental communities, since the diversity linked to the latter samples will be significantly higher than in the tested mock communities. Despite this increased complexity, running IPED on real biological samples still showed a clear improvement, which was visualized using rarefaction curves showing a clear decrease in the number of OTUs. Moreover, a more accurate correlation was found between biologically related samples when comparing the OTU tables produced where IPED was integrated into the workflow, as shown in the results by producing denser clusters distinguishing two different biological conditions.

Where Pre-cluster and UNOISE have an impressive speed, IPED needs more calculation time due to the machine learning classifier required in the first step of IPED. However, as seen in the results, IPED led to a more pronounced improvement in accuracy compared to both of them.

At this stage we only tested our IPED algorithm on mock datasets containing paired end reads that are at least partially overlapping, or in some cases completely overlapping. Recent papers suggest the usage of primer pairs for amplicon sequencing producing paired-end reads which are not overlapping at all, as this approach allows flexibility in development of PCR primers and selection of the hypervariable regions. This way those primers can be selected that allow the most optimal distinction for a specific type of sample [34]. Within this area IPED can have a more pronounced effect on the final results as our algorithm was shown to be most effective in the non-overlapping part of the second read, which in such a case would mean the complete second read. IPED has only been tested on 16S rRNA gene amplicon sequencing data. In principle our tool can be used for any amplicon sequencing data set, such as 18S rRNA, 23S rRNA or 28S rRNA, whenever a reliable reference alignment dataset is available. IPED was developed to be applied after the mothur make.contigs command; yet, further adjustments are needed to make it compatible with other paired-end assemblers. Preliminary data showed that IPED was able to reduce the error rate of QIIME to the same extent.

## Conclusion

We have presented in this work the denoising algorithm IPED specifically developed for Illumina MiSeq 16S rRNA gene amplicon sequencing data. IPED obtains a better performance on mock datasets compared with the available alternatives Pre-cluster and UNOISE, and on average can correct double the amount of errors compared to both algorithms. The beneficial effect of this improved denoising was reflected in more accurate OTU clustering results.

### Ethics approval and consent to participate

Not applicable.

### Consent for publication

Not applicable.

### Availability of data and materials

Four publicly available Illumina MiSeq sequencing datasets of mock communities were used within this work. The first mock community (MOCK1) is available at http://www.mothur.org/MiSeqDevelopmentData.html as presented in Kozich et al. [5]. The second mock community (MOCK2) is available in the EBI European Nucleotide Archive under the project ID PRJEB4688, and is presented in the work of Nelson et al. [22]. The third mock community (MOCK3) is available at the NCBI Sequence Read Archive under the accession number SRP066114. The fourth mock community (MOCK4) is available on the European Nucleotide Archive under the project number PRJEB6244 as presented in Schirmer et al. [11].

The software developed within this work (IPED) is available via github (https://github.com/M-Mysara/IPED) or via http://science.sckcen.be/en/Institutes/EHS/MCB/MIC/Bioinformatics/, including documentation and a tutorial video.

## Additional file

Additional file 1: Extra information describing the development of the machine learning implementation (training and testing), pre-processing of the sequencing data, computational costs and comparative analyses. (DOCX 3212 kb)

## Abbreviations

IPED: Illumina MiSeq Paired-End Denoiser
OTU: Operational Taxonomic Unit
TP: true positives
FN: false negatives
TN: true negatives
FP: false positives
MCC: Mathews Correlation Coefficient
ROC: receiver operating characteristics
MLP: multilayer perceptron
AUC: area under the curve
PCoA: principle coordinate analysis
