# Supplementary material for: IPED: a highly efficient denoising tool for Illumina MiSeq Paired-end 16S rRNA gene amplicon sequencing data
Source: BMC Bioinformatics. 2016 Apr 29;17:192. doi: 10.1186/s12859-016-1061-2 (PMC4850673; doi:10.1186/s12859-016-1061-2)
Supplement: Additional file 1: — Extra information describing the development of the machine learning implementation (training and testing), pre-processing of the sequencing data, computational costs and comparative analyses. (DOCX 3212 kb) [file 12859_2016_1061_MOESM1_ESM.docx]

**Supplementary material for "IPED: A Highly Efficient Denoising Tool for Illumina MiSeq Paired-end Amplicon Sequencing Data"**

Here we provide additional information regarding the construction of the training and testing datasets, building the classifier, evolution of the error rates and effect of denoising tools on the Operational Taxonomic Unit (OTU) analysis.

Contents

[**1.** **From Sequencing Data of a Mock community to Training Dataset** 1](#_Toc438152277)

[1.1. Attribute Selection 3](#_Toc438152278)

[1.2. Selecting The Optimal Ratio between Error:Non-error Instances. 4](#_Toc438152279)

[RandomForest 4](#_Toc438152280)

[**2.** **Classifier Building** 6](#_Toc438152281)

[2.1. Different Classifiers Involved 6](#_Toc438152282)

[2.2.1. MultilayerPerceptpron (MLP) 6](#_Toc438152283)

[2.2.2. Support Vector Machine (SMO) 6](#_Toc438152284)

[2.2.3. Nearest Neighborhood (IBK) 6](#_Toc438152285)

[2.2.4. Logistic 6](#_Toc438152286)

[2.2.5. RandomForest 6](#_Toc438152287)

[2.3. Parameters Configuration 7](#_Toc438152288)

[2.4. Default Configuration Performance 8](#_Toc438152289)

[2.5. Default versus Different Parameter Configuration 9](#_Toc438152290)

[2.6. Learning Curves 11](#_Toc438152291)

[2.7. Voting Results 12](#_Toc438152292)

[**3.** **Pre-Processing Sequencing Data** 14](#_Toc438152293)

[**4.** **Different Paired-End Assemblers** 15](#_Toc438152294)

[**5.** **MOCK4 Data Analysis** 18](#_Toc438152295)

[**6.** **Error Rate Over Different Read Positions** 20](#_Toc438152296)

[**7** **Computational Cost Analysis** 22](#_Toc438152297)

[**8** **Denoising Effect on Operational Taxonomic Units (OTUs) Clustering** 23](#_Toc438152298)

[8.1 Assessment of OTUs Numbers After Denoising 23](#_Toc438152299)

[8.2 Assessment of OTUs Alpha Diversity using Rarefaction Curves 24](#_Toc438152300)

[8.3 Assessment of the Impact of the Denoising Algorithms on the Clustering Quality 27](#_Toc438152301)

[8.4 Assessment of the Influence of Denoising Algorithms on the OTU Clustering Quality 28](#_Toc438152302)

[**9** **Denoising Effect on a Real-Life Biological Samples** 30](#_Toc438152303)

[**10** **Effect of presence of chimera and alignment reference database** 33](#_Toc438152304)

[**11** **References** 34](#_Toc438152305)

1. **From Sequencing Data of a Mock community to Training Dataset**

In this work two publicly available Illumin MiSeq sequencing datasets of mock communities were used as presented in Kozich et al. [1] and Nelson *et al.* [2]. Merging both reads into one contig will result in different contig lengths leading to a length of 250 bp lengths (completely overlapping paired reads) for V4 in both mock communities, lengths of 375 nt and 390 nt for V45 in MOCK1 and MOCK2 respectively and a length of 430 for V34 in MOCK1. Each region in MOCK1 consists of four samples named (130401, 130403, 130417, and 130422) leading to 12 samples for the three variable regions. In MOCK2, sequencing data for each region (V4 & V45) were prepared in duplicate (named v4.I.1, v4.I.05, v4.v5.I.1 & v4.v5.I.11 respectively). In addition, a third mock community - called MOCK3 – was used, which is described in detail in section 4 of the supplementary material.

As training data, 3,000 reads were randomly selected from sample 130401 of MOCK1 (1,000 reads from the V34, V4 and V45 regions respectively). Important to notice is that all three samples used during the training process are completely disregarded in the subsequent benchmarking analysis. Each nucleotide in those reads was evaluated as being erroneous (mismatch, insertion or deletion) or correct based on a aligning those reads against the reference genomes using a combination of Blast [3] and ClustalW [4].

This lead to a training dataset consisting of 1,031,625 instances representing nucleotide positions. This training data was cleaned as follows: dereplication (668,962 remaining), removal of outliers based on interquartile ranges (remainder of 664,301), randomization, and simplification via selecting subset of the features [see below]. Next, the data are split into three folds preserving the same ratio between errors:non-errors instances throughout the three folds: (a) a learning data set for training the classifier, (b) a validation set for selecting the most optimal classifier and (c) a testing data set for final validation. Subset (a) was further modified by adjusting the ratio between erroneous and non-erroneous instances [see below], and for subset (b) we used an equal ratio between both classes, while subset (c) was kept unchanged.

## Attribute Selection

"*Feature subset selection is the process of identifying and removing as much irrelevant and redundant information as possible. This reduces the dimensionality of the data and may allow learning algorithms to operate faster and more effectively. In some cases, accuracy on future classification can be improved; in others, the result is a more compact, easily interpreted representation of the target concept*" [5].

Feature selection is performed using a combination of two algorithms i.e. a searching algorithm and an evaluation algorithm. Here we applied a search algorithm named "best first search" that allows backtracking along the searching process via moving through the search space making an addition or a deletion of a single feature subset at a time. In the occasions where the search path leads to a less promising subset of features, it would recall a more promising subset and continue searching from there. Subsets are evaluated via an evaluation algorithm named "CfsSubsetEval " that allows identification of those features having a high predictive power for the class (distinguishing erroneous positions from correct positions) via selecting subset of feature that have a high correlation with the class label, while having a low intercorrelation with other predictive features. Here we use three folds cross validation to select the minimum set of feature with a high capability to distinguish erroneous instances from a total of 17 features.

Supplementary table 1 Illustration of attributes selection via three fold cross validation results, where 100% means the attribute was found worthy in the three tests, 0 means it was found unworthy in the three tests. (three folds cross validation).

| **Forward attributes** | **Percentage** | **Reverse attributes** | | **Percentage** |
| --- | --- | --- | --- | --- |
| Position number | 100 | Position number | | 33 |
| Succeeding position homopolymer | 0 | Succeeding position homopolymer | | 0 |
| Position homopolymer | 33 | Position homopolymer | | 0 |
| Proceeding position homopolymer | 0 | Proceeding position homopolymer | | 0 |
| Succeeding position Phred | 0 | Succeeding position Phred | | 0 |
| Position Phred | 100 | Position Phred | | 100 |
| Proceeding position Phred | 0 | Proceeding position Phred | | 0 |
| Motif marker | 0 | Motif marker | | 0 |
| **Contig attribute** | | | Percentage | |
| Relation between forward and reverse calling | | | 100 | |

## Selecting The Optimal Ratio between Error:Non-error Instances.

There is a skewed class distribution in the complete training data set as the ratio between error : non-error instances is 1 : 45. Machine learning classifiers have difficulties when dealing with imbalanced data [6], as they would obtain the maximum accuracy when they would skew their prediction towards the majority class (in this case the non-erroneous instances). Intuitively, we want the classifier also to predict the minority class (i.e. erroneous instances), hence we needed to modify the distribution between both classes. To avoid overfitting, we applied the under-sampling technique, were instances from the majority class are randomly removed to get a reasonable ratio between both classes. We used the Weka supervised instance preprocessing named "SpreadSubsample". To select the correct distribution between error: non-error, we tried several ratios of 1:1, 1:2, 1:3, 1:4 and 1:5 for Error versus Non-error instances respectively and trained various classifiers using each ratio (using their default parameters). Keeping in mind the specificity level in order not to compromise the specificity of the clustering step (i.e. the second step in the IPED algorithm), we selected the ratio 1:3 [error:non-error] as it has it the highest performance ratio for most of the classifiers still preserving an acceptable percentage of specificity. [For your information, for support vector machine classifier, the default parameters shown in the table 2 are not the best performing setting as will be described in the next section of the supplementary material]

Supplementary table 2 Illustration of different classifiers performance upon being trained using various ratios of errors : non-errors instances.

| RandomForest | | | | | | | | | | |
| --- | --- | --- | --- | --- | --- | --- | --- | --- | --- | --- |
| Ratio | | 1:1 | 1:2 | | | 1:3 | 1:4 | | | 1:5 |
| Sensitivity | | 0.77 | 0.66 | | | 0.59 | 0.54 | | | 0.50 |
| Specificity | | 0.78 | 0.86 | | | 0.91 | 0.92 | | | 0.93 |
| ROC | | 0.84 | 0.84 | | | **0.85** | 0.84 | | | 0.84 |
| **Multilayer Perceptron (MLP)** | | | | | | | | | | |
| Ratio | | 1:1 | 1:2 | | 1:3 | | 1:4 | 1:5 | | |
| Sensitivity | | 0.84 | 0.43 | | 0.44 | | 0.32 | 0.35 | | |
| Specificity | | 0.62 | 0.92 | | 0.92 | | 0.96 | 0.96 | | |
| ROC | | 0.81 | 0.81 | | **0.82** | | 0.81 | 0.81 | | |
| **Support Vector Machine (SMO)** | | | | | | | | | | |
| Ratio | | 1:1 | 1:2 | | 1:3 | | 1:4 | 1:5 | | |
| Sensitivity | | 0.65 | 0.23 | | 0.01 | | 0.01 | 0.01 | | |
| Specificity | | 0.68 | 0.94 | | 0.99 | | 0.99 | 0.99 | | |
| ROC | | 0.67 | 0.59 | | 0.51 | | 0.51 | 0.51 | | |
| **logistic** | | | | | | | | | | |
| Ratio | | 1:1 | 1:2 | | 1:3 | | 1:4 | 1:5 | | |
| Sensitivity | | 0.64 | 0.35 | | 0.18 | | 0.09 | 0.04 | | |
| Specificity | | 0.66 | 0.90 | | 0.96 | | 0.98 | 0.99 | | |
| ROC | | 0.72 | 0.718 | | **0.72** | | 0.717 | 0.71 | | |
| Nearest Neighborhood (IBK) | | | | | | | | | | |
| Ratio | 1:1 | | 1:2 | 1:3 | | | 1:4 | | 1:5 | |
| Sensitivity | 0.72 | | 0.64 | 0.58 | | | 0.52 | | 0.48 | |
| Specificity | 0.72 | | 0.82 | 0.86 | | | 0.89 | | 0.91 | |
| ROC | 0.75 | | 0.71 | **0.758** | | | 0.757 | | 0.755 | |


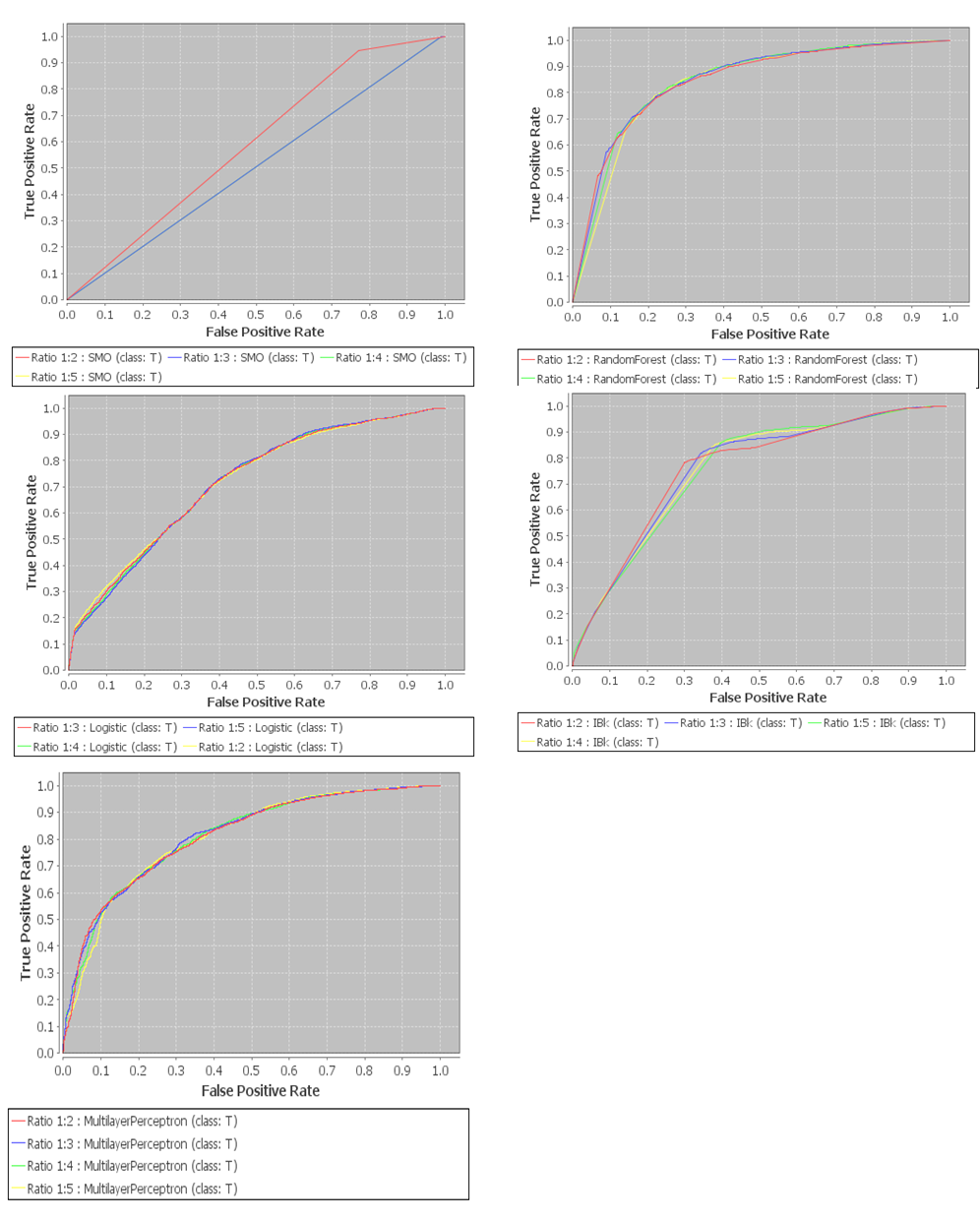


Supplementary figure 1 Plotting various ROC curves of different classifiers performance upon being trained using various ratios of errors : non-errors instances.

1. **Classifier Building**

## Different Classifiers Involved

Different classifiers were trained and subsequently tested on the selected training data set. Here we describe the different classifiers that were considered.

## MultilayerPerceptpron (MLP)

A multilayer perceptron is a backpropagation artificial neural network model. It strength lies in its capability to distinguish non-linearly separable or separable by hyper plane instances. Similar to human brain neurons, it uses a hidden perceptron layer(s) connected to both the input data and the output data. Through the training, a weighting factor is adjusted to each perceptron that fires after reaching the adjusted threshold.


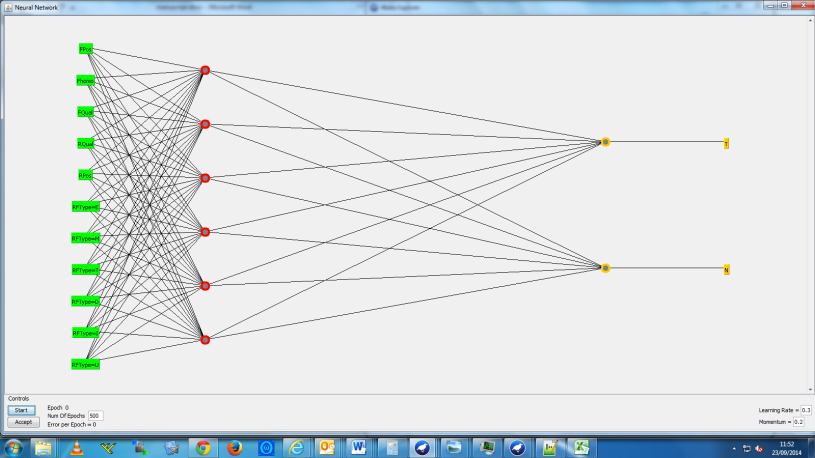


Supplementary figure 2 Illustration of the hidden layer and input and output perceptron used for the training of MLP

## Support Vector Machine (SMO)

SMO is the WEKA implementation of the supervised learning function-based method. It consists of two phases: first, the kernel divides non-linear data into multi-dimensional vectors. Secondly, it break them down into a two dimensional problem to be linearly separable (via an improved version of John Platt's sequential minimal optimization algorithm).

## Nearest Neighborhood (IBK)

IBK (instance based kernel) is a K-nearest neighbor predictor. It finds the K nearest neighbors of that instance and then classifies the item according to the majority of neighboring items

## Logistic

A classifier for building and using a multinomial logistic regression model with a ridge estimator. It estimates the class probabilities and applies a log likelihood to these probabilities.

## RandomForest

Random forest that uses an ensemble of classification unpruned trees (combining both bagging and random variable selection) yields an ensemble that can achieve both low bias and low variance. Each of the classification trees is built using a bootstrap sample of the data, and for each split the candidate set of variables is a random subset of the variables.

## Parameters Configuration

Supplementary table 3 List the different parameters configurations for each classification algorithms included in our analysis, together with the default value to each parameter.

| Classifier | Parameters | Description | Value |
| --- | --- | --- | --- |
| MultilayerPerceptron  (MLP) | hiddenLayers | his defines the hidden layers of the neural network. | a* |
|  | learningRate | The amount the weights are updated | 0.3 |
|  | Momentum | Momentum applied to the weights during updating. | 0.2 |
|  | nominalToBinaryFilter | This will preprocess the instances with the filter. | True |
|  | normalizeAttribute | This will normalize the attributes.(nominal or numeric) | True |
|  | normalizeNumericClass | This will normalize the class if it's numeric between -1 and 1. | True |
|  | Reset | This will allow the network to reset with a lower learning rate. | True |
|  | Seed | Seed used to initialise the random number generator. | 0 |
|  | trainingTime | The number of epochs to train through. | 500 |
|  | validationThreshold | Used to terminate validation testing (number of sequential worse error). | 20 |
| Support Vector Machine  (SMO) | Build logistic model | Whether to fit a logistic model to the outputs | FALSE |
|  | C | Complexity parameter C | 1.0 |
|  | Epsilon | Epsilon for round-off error | 1.0E-12 |
|  | filterType | Determines how/if the data will be transformed. | Normalize |
|  | Kernel | The kernel to us | BUK |
|  | numFolds | The number of folds for cross-validation used to generate training data for logistic models | -1 |
|  | RandomSeed | Random number seed for the cross-validation | 1 |
|  | toleranceParameter | The tolerance parameter | 0.001 |
| Nearest Neighborhood  (IBK) | KNN | The number of neighbours to use. | 1 |
|  | meanSquared | Whether the mean squared error is used rather than mean absolute error | False |
|  | NearestNeighbourSearch | he nearest neighbour search algorithm to use | LinearNN |
|  | windowSize | Gets the maximum number of instances allowed in the training pool. | 0 |
| Logistic | maxIts | Maximum number of iterations to perform. | -1 |
|  | Ridge | Set the Ridge value in the log-likelihood. | 1.0E-8 |
|  | useConjugateGraientD. | Use conjugate gradient descent rather than BFGS updates; | False |
| RandomForest | maxDepth | The maximum depth of the trees, 0 for unlimited. | 0 |
|  | numExecutionSlots | The number of execution slots (threads) to use for constructing the ensemble. | 1 |
|  | numFeatures | The number of attributes to be used in random selection. | 0 |
|  | numTrees | The number of trees to be generated. | 10 |
|  | Seed | The random number seed to be used. | 1 |

* 'a' = (number of attributes +number of classes) / 2.

## Default Configuration Performance

To test the performance of each of the considered classifiers using their default settings, we used the learning set (subset "a") for training the classifiers and validation set (subset "b") for testing the classifiers. Random Forest was found to have the highest performance

Supplementary table 4 Performance of each classifiers trained using learning set (subset a) and tested using validation set (subset b) regarding specificity, sensitivity, MCC and ROC.

| single tools | Specificity | Sensitivity | MCC | ROC |
| --- | --- | --- | --- | --- |
| MLP | 92 | 44 | 42 | 82 |
| SMO | 1 | 99.9 | 7 | 51 |
| IBK | 86 | 58 | 46 | 76 |
| Logistic | 96 | 18 | 22 | 72 |
| RandomForest | 91 | 59 | 52 | 85 |

## Default versus Different Parameter Configuration

Several parameter settings have been tested for the selected classifiers (implemented in WEKA). Here we show the performance of each classifier evaluated via the Matthews Correlation Coefficient (MCC) upon testing using the TrainingDB subset (b) after training using the TrainingDB subset (a). MCC was used as criterion as it considers both sensitivity and specificity of the classifier (kindly refer to the main manuscript for the exact definitions as well as equations). We show the default parameter setting (config 1) compared to adjusted parameter settings (leading to different classifiers) as shown in table 5. In figure 3, we plot the MCC of each configuration per classifier.

Supplementary table 5 Evaluation of different parameter configurations for various classifiers; refer to table 3 for description of the default parameters.

| Configuration | **MLP*** | **SMO** | **IBK** | **Logistic** | **RandomForest** |
| --- | --- | --- | --- | --- | --- |
| 1 | default | default | default | default | default |
| 2 | Learning rate = 0.1 | Build logistic model=True | KNN=2 | Ridge=  1.0E-4 | maxDepth=1 |
| 3 | Momentum= 0.1 | C=2 | meanSuared=True | Ridge=  1.0E-8 | numExcution Slots= 3 |
| 4 | Learning rate = 0.5 | epsilon = 1.0E-8 | SearchAlgorithm= KDTree | useConjugateGraientD= True | numTree=12 |
| 5 | Momentum= 0.4 | Kernel= RBFKernel. | SearchAlgorithm= BallTree | - | numTree=8 |
| 6 | TrainingTime=5000 | Kernel= PUK. | SearchAlgorithm= CoverTree | - | numFeature= 1 |
| 7 | validationThreshold=10 | Conf 6 + 2 | Windowsize=1 | - | Seed=2 |
| 8 | validationThreshold=30 | Conf 6 + 3 | - | - | - |
| 9 | hiddenLayers=11 | Conf 6 + 4 | - | - | - |
| 10 | hiddenLayers=2 | - | - | - | - |
| 11 | hiddenLayers=6,4 | - | - | - | - |
| 12 | Conf 2 + 3 | - | - | - | - |
| 13 | Conf 4 + 5 | - | - | - | - |
| 14 | Conf 4 + 5 + 11 | - | - | - | - |

*** For MLP we tried more combinations, here we only shows some of them.**

To compare the different parameters setting, we used the MCC that combines both specificity and sensitivity, using the validation set (subset "b") for testing, and learning set (subset "a" for training). The results revealed no significant improvement gained from the default setting with exception of SMO (PUK kernel was shown to obtain better results than the default setting).

Supplementary figure 3 Plot showing Matthews Correlation Coefficient score of various parameter setting, for Logistic regression, SMO, MLP, IBK and RandomForest. The default configuration is numbered 1, while different configurations are numbered as shown in table 5.

## Learning Curves

To assess the size of the training data, we plotted the learning curves for each classifier. The performance is evaluated by the percentage of correctly predicted instances, and we used the validation set (subset "b") for evaluation (i.e. a dataset with an equal ratio between Error/Non-Error instances), and the learning set (subset "a") for training. From the graph (Fig 4) we noticed that the classifiers reach their maximum performance upon exceeding 50% of the size of the learning set (subset "a").

Supplementary figure 4 Learning curve showing different sizes of the training dataset (TrainDB subset a) in percentages, where 100 means using the whole data, and 1 means using 1% of the data.

## Voting Results

Next we tried combining those classifiers using an ensemble approach called voting. In voting, each of the selected classifiers (base level classifiers) outputs a probability distribution vector and the class with the highest average probability over all classifiers is selected as the ensemble output for the respective test instance. Voting approaches considering two, three, four or five classifiers were tested. The voting classifier combining MLP and Random Forest was found to have the highest accuracy compared to other combinations as shown in table 6. Compared to each individual tool, it had the highest area under the curve (AUC) as seen in figure 5.


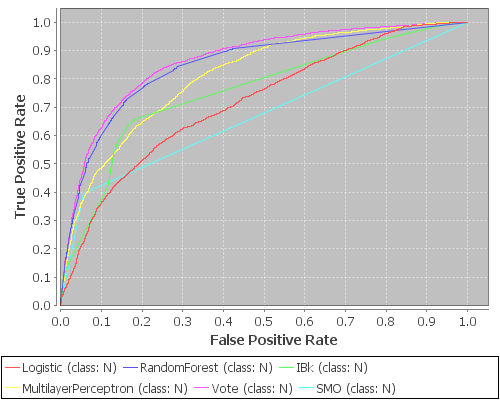


Supplementary figure 5. ROC curve showing the performance of the different individual classifiers as well as the best performing voting combination, existing of MLP and RandomForest to test the sensitivity (Y axes) and 1- specificity (X axes). The area under the curve (AUC) was found to be highest with the voting classifier, achieving 0.87, compared to the second best classifier (RandomForest), with AUC equals 0.85.

Supplementary table 6 Illustration of the performance of each two-classifier voting approach via specificity, sensitivity, MCC and ROC. The best performing combination is highlighted in red.

|  | Specificity | | | | |
| --- | --- | --- | --- | --- | --- |
|  | MLP | SMO | IBK | Logistic | Random  Forest |
| MLP | - | 94 | 86 | 95 | **93** |
| SMO | - | - | 94 | 94 | 95 |
| IBK | - | - | - | 87 | 86 |
| Logistic | - | - | - | - | 94 |
| RandomForest | - | - | - | - | - |
|  | Sensitivity | | | | |
|  | MLP | SMO | IBK | Logistic | Random  Forest |
| MLP | - | 40 | 61 | 35 | **57** |
| SMO | - | - | 40 | 40 | 39 |
| IBK | - | - | - | 57 | 60 |
| Logistic | - | - | - | - | 48 |
| RandomForest | - | - | - | - | - |
|  | MCC | | | | |
|  | MLP | SMO | IBK | Logistic | Random  Forest |
| MLP | - | 41 | 48 | 37 | **53** |
| SMO | - | - | 41 | 41 | 41 |
| IBK | - | - | - | 45 | 48 |
| Logistic |  | - | - | - | 46 |
| RandomForest |  | - | - | - | - |
|  | ROC | | | | |
|  | MLP | SMO | IBK | Logistic | Random  Forest |
| MLP | - | 82 | 83 | 80 | **87** |
| SMO | - | - | 78 | 74 | 85 |
| IBK | - | - | - | 80 | 84 |
| Logistic | - | - | - | - | 86 |
| RandomForest | - | - | - | - | - |

Supplementary table 7 Illustration of the performance of each three-classifier voting approach via specificity, sensitivity, MCC and ROC.

| Cobmination | | | Specificity | Sensitivity | MCC | ROC |
| --- | --- | --- | --- | --- | --- | --- |
| MLP | SMO | IBK | 94 | 43 | 43 | 84 |
| MLP | SMO | Logistic | 95 | 40 | 41 | 81 |
| MLP | SMO | RandomForest | 94 | 41 | 42 | 86 |
| MLP | IBK | Logistic | 90 | 53 | 47 | 83 |
| MLP | IBK | RandomForest | 88 | 59 | 49 | 86 |
| MLP | Logistic | RandomForest | 95 | 45 | 46 | 86 |
| SMO | IBK | Logistic | 95 | 40 | 41 | 81 |
| SMO | IBK | RandomForest | 92 | 55 | 51 | 85 |
| SMO | Logistic | RandomForest | 95 | 39 | 41 | 86 |
| IBK | Logistic | RandomForest | 89 | 57 | 48 | 85 |

Supplementary table 8 Illustration of the performance of each four-classifier voting approach via specificity, sensitivity, MCC and ROC.

| Cobmination | | | | Specificity | Sensitivity | MCC | ROC |
| --- | --- | --- | --- | --- | --- | --- | --- |
| MLP | SMO | IBK | Logistic | 95 | 40 | 42 | 83 |
| MLP | SMO | IBK | RandomForest | 93 | 53 | 51 | 86 |
| MLP | SMO | Logistic | RandomForest | 95 | 40 | 41 | 86 |
| MLP | IBK | Logistic | RandomForest | 91 | 55 | 49 | 85 |
| SMO | IBK | Logistic | RandomForest | 94 | 49 | 48 | 85 |

Supplementary table 9 Illustration of the performance of all five-classifiers voting approach via specificity, sensitivity, MCC and ROC.

|  |  |  |  |  | Specificity | Sensitivity | MCC | ROC |
| --- | --- | --- | --- | --- | --- | --- | --- | --- |
| MLP | SMO | IBK | Logistic | RandomForest | 95 | 46 | 46 | 86 |

1. **Pre-Processing Sequencing Data**

The sequencing data (MOCK1, MOCK2 and MOCK3) were trimmed, aligned, screened, filtered and dereplicated using the mothur [7] software package (v.1.33.3), via the following commands:

1. Contigs were formed by merging the paired-end reads using the heuristic based on the difference in Phred quality scores of both reads as proposed in Kozich et al. [1] using the mothur (make.contigs command). This command will extract the sequence and quality score data from fastq files, create the reverse complement of the reverse read and then join the reads into contigs. Next, based on the alignments, those positions are identified where the two reads disagree. If one sequence has a base and the other has a gap, the quality score of the base must be over 25 to be considered real. If both sequences have a base at that position, then we require one of the bases to have a quality score 6 or more points better than the other. If it is less than 6 points better, then we set the consensus base to an N.
2. Contigs were culled if they had an ambiguous base, or they had a length exceeding the expected lengths (250 nt for V4 MOCK1 and MOCK2, 375 nt for V45 MOCK1, 390 nt for V45 MOCK2, 430 nt for V34 MOCK1) using mothur (trim.seqs command).
3. Reads not aligning the correct regions were removed using (screen.seqs command).
4. Afterwards, reads are dereplicated, using the mothur (unique.seqs command).
5. Denoising is performed using either Pre-cluster (pre.cluster command) or IPED. In addition, a non-denoised version of the sample was kept for further analysis.
6. To have an idea on the beneficial effect of IPED over Pre-cluster in the downstream analysis, we performed OTU-clustering on the MOCK1 and MOCK2 datasets. Creating operational taxonomic unit (OTU) was performed using both average neighborhood clustering approach as implemented in mothur (dist.seqs command preceded by the cluster command) and UPARSE [8] (v7.0.1001_i86linux32 – commands sortbysize, cluster_otus, and usearch_global) with default parameters settings, with exception of singleton removal for UPARSE. The default setting of singleton removal was deactivated to accurately assess the effect of sequencing errors on all the OTUs produced, including singletons. Other statistical analyses were performed using mothur (rarefaction.single, pcoa and summary.seqs commands).
7. **Different Paired-End Assemblers**

A comparative analysis was performed to assess the differences between various paired-end assembly tools (and their quality trimming) used for amplicon sequencing implemented in mothur, QIIME andUPARSE, together with PEAR.:

1. Mothur [7]: Contigs were formed by merging the paired-end reads using the heuristic based on the difference in Phred quality scores of both reads as proposed in Kozich et al. [1] using the mothur (make.contigs command). This command will extract the sequence and quality score data from fastq files, create the reverse complement of the reverse read and then join the reads into contigs. Next, based on the alignments, those positions are identified where the two reads disagree. If one sequence has a base and the other has a gap, the quality score of the base must be over 25 to be considered real. If both sequences have a base at that position, then we require one of the bases to have a quality score 6 or more points better than the other. If it is less than 6 points better, then we set the consensus base to an N. This was done using mothur (v.1.33.3).
2. QIIME [9]: The paired-end reads were joined using the join_paired_ends command. Next, those contigs are subjected to quality trimming using Phred/Phrap quality scores associated with each base to trim sequence reads (split_libraries_fastq command) , as described in details in Bokulich et al [10]. This was done using using QIIME (v 1.8.0).
3. UPARSE pipeline [8] (as implemented in USEARCH): The paired-end reads were joined using the fastq_mergepairs command. Next those contigs are subjected to quality trimming using the fastq_filter command applying a maximum expected error threshold.
4. PEAR [11] is designed to maximize assembly scores (handling mismatch or ambiguity conflicts between both paired-end reads) leading to higher number of assembled reads. The program evaluates all possible paired-end read overlaps and does not require the target fragment size as input. It also implements a statistical test for minimizing false-positive results.

All paired-end assembly algorithms, were applied on the MOCK1 and MOCK2 datasets. Reads with ambiguous bases or homopolymers longer than 8 nucleotides were removed, chimeras were identified and subsequently removed, and the error rate was reported (seq.error command in mothur) using the full length 16S rRNA genes of the mock community species as reference. OTU-clustering was performed using UPARSE [8] (v7.0.1001_i86linux32 – commands sortbysize, cluster_otus, and usearch_global).

Following the pipeline outlined above, we compared the paired-end assembly tools as implemented in mothur, QIIME and USEARCH as well as the PEAR software, by applying each of them on the MOCK1, MOCK2 and MOCK3 datasets. Despite the fact that those assembly algorithms cannot be seen as denoising algorithms in a strict sens, applying them will have an effect on the error rate. Indeed, as each of these assembly algorithms includes or is proceeded by quality checking steps, we also assessed their effect on the error rate, number of OTUs and number of assembled reads. For this analysis, fastq_mergepairs (USEARCH) and mothur (make.contigs), join_paired_ends (QIIME) and PEAR achieved an error rate of 0.0027, 0.0029, 0.0031 and 0.0097, respectively. Those error rates are still significantly higher than the error rate obtained with applying IPED on the output of the mothur make.contigs command, i.e. 0.0010.

Supplementary table 10 Comparative study between various paired-end merger including PEAR, fastq_mergepairs (USEARCH), join_paired_ends (QIIME) and make.contigs (mothur), including the number of assembled reads and error rate and the number of OTUs produced after clustering using both cluster (mothur) and cluster_otus (UPARSE). In addition, we also report the corresponding results for make.contigs after being denoised with IPED. We did not report the values when it is not feasible to calculate the OTUs, nor the numbers are over-estimated as in MOCK1 V45.

| mock name | Region | ID | **% reads rejected from the original reads** | | | | **Error Rate** | | | | |
| --- | --- | --- | --- | --- | --- | --- | --- | --- | --- | --- | --- |
|  |  |  | **PEAR** | **USEARCH** | **QIIME** | **Mothur** | **PEAR** | **USEARCH** | **QIIME** | **Mothur** | **Mothur + IPED** |
| **MOCK1** | **V34** | 130403 | 7 | 98 | 52 | 69 | 0.0111 | 0.0017 | 0.0034 | 0.0026 | 0.0002 |
|  |  | 130417 | 7 | 98 | 52 | 69 | 0.0097 | 0.0017 | 0.0029 | 0.0023 | 0.0003 |
|  |  | 130422 | 6 | 97 | 36 | 48 | 0.0075 | 0.0019 | 0.0030 | 0.0028 | 0.0008 |
|  | **V4** | 130403 | 7 | 30 | 84 | 41 | 0.00707 | 0.00096 | 0.00200 | 0.00056 | 0.00010 |
|  |  | 130417 | 7 | 22 | 34 | 31 | 0.00417 | 0.00078 | 0.00136 | 0.00051 | 0.00008 |
|  |  | 130422 | 6 | 16 | 11 | 22 | 0.00282 | 0.00071 | 0.00116 | 0.00049 | 0.00008 |
|  | **V45** | 130403 | 10 | 79 | 64 | 66 | 0.0334 | 0.0049 | 0.0075 | 0.0084 | 0.0022 |
|  |  | 130417 | 11 | 72 | 63 | 63 | 0.0241 | 0.0047 | 0.0062 | 0.0069 | 0.0020 |
|  |  | 130422 | 10 | 57 | 40 | 43 | 0.0169 | 0.0042 | 0.0060 | 0.0060 | 0.0016 |
| **MOCK2** | **V4** | v4.I.1 | 16 | 17 | 17 | 6 | 0.00080 | 0.00071 | 0.00079 | 0.00061 | 0.00004 |
|  |  | v4.I.05 | 20 | 21 | 20 | 6 | 0.00088 | 0.00078 | 0.00087 | 0.00068 | 0.00004 |
|  | **V45** | v4.v5.1 | 27 | 69 | 44 | 16 | 0.0124 | 0.0082 | 0.0072 | 0.0066 | 0.0041 |
|  |  | v4.v5.I.11 | 28 | 35 | 45 | 4 | 0.0092 | 0.0085 | 0.0034 | 0.0033 | 0.0031 |
| **MOCK3** | **V34** | M1 | 21 | 47 | 58 | 43 | 0.0054 | 0.0014 | 0.0014 | 0.0015 | 0.0004 |
|  |  | M2 | 22 | 49 | 60 | 52 | 0.0053 | 0.0014 | 0.0014 | 0.0015 | 0.0006 |
|  |  | M3 | 20 | 47 | 88 | 51 | 0.0051 | 0.0013 | 0.0011 | 0.0014 | 0.0005 |
| **Average** | | | **14** | **53** | **48** | **39** | **0.0097** | **0.0027** | **0.0031** | **0.0029** | **0.0010** |
| mock name | ID | **PEAR** | **USEARCH** | **QIIME** | **Mothur** | **Mothur + IPED** | **PEAR** | **USEARCH** | **QIIME** | **Mothur** | **Mothur + IPED** |
| **Clustering algorithm** | | Mothur Clustering (cluster) | | | | | UPARSE clustering (cluster_otus) | | | | |
| **MOCK1** | 130403 | 9245 | 15 | 112 | 58 | 45 | 6894 | 15 | 105 | 51 | 43 |
|  | 130417 | 5705 | 15 | 48 | 52 | 39 | 4167 | 15 | 53 | 45 | 40 |
|  | 130422 | 2798 | 15 | 111 | 83 | 61 | 2168 | 15 | 57 | 71 | 55 |
|  | 130403 |  | 204 | 665 | 165 | 135 | 53907 | 191 | 1035 | 158 | 124 |
|  | 130417 |  | 168 | 1294 | 142 | 118 | 25755 | 157 | 1279 | 139 | 112 |
|  | 130422 |  | 147 | 852 | 123 | 109 | 14586 | 142 | 1297 | 116 | 102 |
|  | 130403 |  |  |  |  |  |  |  |  |  |  |
|  | 130417 |  |  |  |  |  |  |  |  |  |  |
|  | 130422 |  |  |  |  |  |  |  |  |  |  |
| **MOCK2** | v4.I.1 | 63 | 43 | 1497 | 58 | 55 | 51 | 36 | 77 | 32 | 29 |
|  | v4.I.05 | 68 | 52 | 2156 | 57 | 52 | 50 | 36 | 89 | 31 | 26 |
|  | v4.v5.1 | 185 | 59 | 567 | 54 | 29 | 60 | 23 | 71 | 44 | 25 |
|  | v4.v5.I.11 | 1092 | 669 | 6593 | 200 | 133 | 208 | 33 | 295 | 92 | 46 |
| **MOCK3** | M1 | 1417 | 101 | 97 | 85 | 83 | 1095 | 41 | 30 | 42 | 39 |
|  | M2 | 2388 | 148 | 146 | 123 | 113 | 1862 | 60 | 48 | 55 | 47 |
|  | M3 | 830 | 74 | 40 | 61 | 57 | 654 | 31 | 15 | 33 | 28 |

Important to notice is that these assembly algorithms had a different profile when it comes to the number of assembled reads, where the percentage of reads removed were on average 53%, 39%, 48% and 14% respectively. The different algorithms implemented in each approach as well as the different quality checks proposed by each tool accounted for these differences in the number of assembled reads. Upon investigating the downstream analysis, UPARSE clustering approach was applied on the MOCK1 (V34 and V4 samples) dataset as well as the MOCK2 dataset, and the resulting number of OTUs arereported below after applying rarefication, as described before. The number of OTUs were on average 30, 29, 131, and 192 for fastq_mergepairs, make.contig, join_paired_ends and PEAR respectively. Similar level was reported for MOCK3.

1. **MOCK4 Data Analysis**

We examined the effect of applying IPED on a fourth mock community - called MOCK4 - consisting of 73 samples recently published in Schirmer et al [12]. Their microbial composition ranges from single species to diverse mock communities (49 bacteria and 10 archaea) with either even or uneven abundance distributions. Five different Illumina MiSeq library preparations were used to amplify the V4 and the V34 region. Contigs constructed via merging both reads resulted in different lengths, ranging from 253 nucleotides (i.e. almost completely overlapping reads) to 450 nucleotides (partially overlapping reads). A detailed description of MOCK3 can be found in the original publication. From the results we can observe the same trend in lowering the error rate when applying IPED. Indeed, when both reads are almost completely overlapping (contig length ranging between 253 and 292), IPED was able to reduce the error rate from 0.0041 to 0.0032, while the effect was more prominent when dealing with contigs have a smaller overlap between both reads (contig length ranging between 330 and 450), showing a decrease in the error rate from 0.0065 to 0.0033.

Supplementary table 11 Illustration of the MOCK3 samples, showing the amplified region as well as the average length of the produced reads after pre-processing (See above) together with the error rate with versus without IPED. Sample with metaID 54 was missing and metaID 36, 38 and 48 were excluded as they exceeded 2 million reads per sample (with exceeding 600,000 unique reads).

| **Meta ID** | Region | ave. Length | Without IPED | IPED | **Meta ID** | Region | ave. Length | Without IPED | IPED |
| --- | --- | --- | --- | --- | --- | --- | --- | --- | --- |
| **19** | V4 | 292 | 0.0082 | 0.0078 | **62** | V4 | 292 | 0.0057 | 0.0055 |
| **20** | V4 | 292 | 0.0081 | 0.0076 | **64** | V4 | 292 | 0.0058 | 0.0057 |
| **21** | V4 | 292 | 0.0081 | 0.0078 | **65** | V4 | 292 | 0.0056 | 0.0055 |
| **22** | V4 | 292 | 0.0082 | 0.0075 | **66** | V4 | 292 | 0.0059 | 0.0058 |
| **23** | V4 | 292 | 0.0077 | 0.0050 | **67** | V4 | 292 | 0.0057 | 0.0055 |
| **24** | V4 | 292 | 0.0077 | 0.0054 | **68** | V4 | 292 | 0.0048 | 0.0042 |
| **25** | V4 | 292 | 0.0077 | 0.0046 | **69** | V4 | 292 | 0.0047 | 0.0037 |
| **26** | V4 | 292 | 0.0078 | 0.0056 | **71** | V4 | 292 | 0.0046 | 0.0035 |
| **27** | V3/V4 | 275 | 0.0053 | 0.0042 | **74** | V4 | 292 | 0.0061 | 0.0058 |
| **28** | V3/V4 | 275 | 0.0041 | 0.0040 | **75** | V4 | 292 | 0.0058 | 0.0056 |
| **29** | V3/V4 | 275 | 0.0024 | 0.0017 | **76** | V4 | 292 | 0.0058 | 0.0000 |
| **30** | V3/V4 | 275 | 0.0046 | 0.0047 | **77** | V4 | 253 | 0.0013 | 0.0008 |
| **31** | V3/V4 | 275 | 0.0047 | 0.0043 | **78** | V4 | 253 | 0.0020 | 0.0017 |
| **32** | V3/V4 | 275 | 0.0032 | 0.0030 | **79** | V4 | 253 | 0.0019 | 0.0016 |
| **33** | V3/V4 | 275 | 0.0024 | 0.0015 | **80** | V4 | 253 | 0.0020 | 0.0017 |
| **34** | V3/V4 | 275 | 0.0038 | 0.0036 | **81** | V4 | 253 | 0.0020 | 0.0017 |
| **35** | V4 | 292 | 0.0063 | 0.0046 | **82** | V4 | 253 | 0.0020 | 0.0015 |
| **37** | V4 | 292 | 0.0074 | 0.0056 | **83** | V4 | 253 | 0.0025 | 0.0019 |
| **39** | V4 | 253 | 0.0022 | 0.0018 | **85** | V4 | 253 | 0.0020 | 0.0015 |
| **40** | V4 | 253 | 0.0029 | 0.0025 | **86** | V4 | 292 | 0.0071 | 0.0043 |
| **41** | V4 | 253 | 0.0022 | 0.0019 | **87** | V4 | 292 | 0.0070 | 0.0034 |
| **42** | V4 | 253 | 0.0022 | 0.0017 | **88** | V4 | 292 | 0.0081 | 0.0048 |
| **43** | V4 | 253 | 0.0019 | 0.0014 | **89** | V4 | 292 | 0.0063 | 0.0026 |
| **44** | V4 | 253 | 0.0023 | 0.0015 | **90** | V4 | 292 | 0.0064 | 0.0027 |
| **45** | V4 | 253 | 0.0022 | 0.0014 | **91** | V4 | 292 | 0.0063 | 0.0028 |
| **46** | V4 | 253 | 0.0021 | 0.0014 | **93** | V4 | 292 | 0.0054 | 0.0014 |
| **47** | V4 | 253 | 0.0015 | 0.0009 | **94** | V4 | 292 | 0.0055 | 0.0016 |
| **49** | V4 | 292 | 0.0065 | 0.0034 | **96** | V4 | 292 | 0.0057 | 0.0018 |
| **50** | 16S | 330 | 0.0017 | 0.0015 | **97** | V3/V4 | 450 | 0.0097 | 0.0056 |
| **51** | 16S | 330 | 0.0056 | 0.0036 | **98** | V3/V4 | 450 | 0.0094 | 0.0045 |
| **52** | V4 | 292 | 0.0051 | 0.0049 | **99** | V3/V4 | 450 | 0.0098 | 0.0049 |
| **53** | V4 | 292 | 0.0057 | 0.0055 | **100** | V3/V4 | 450 | 0.0089 | 0.0037 |
| **59** | V4 | 292 | 0.0063 | 0.0062 | **101** | V3/V4 | 450 | 0.0088 | 0.0039 |
| **60** | V4 | 292 | 0.0061 | 0.0060 | **102** | V3/V4 | 450 | 0.0091 | 0.0039 |
| **61** | V4 | 292 | 0.0063 | 0.0061 |  |  |  |  |  |

It is important to stress that the raw error rate observed for this mock community is higher than what can reasonably be expected from Illumina MiSeq sequencing data. A possible contamination would explain the increase in the overall error rate. Therefore we were cautious in attaching too harsh conclusions to this data set, nor to expand the analysis to the rest of the other approaches.

1. **Error Rate Over Different Read Positions**

We examined the effect of applying both denoising algorithms relative to the position in the read in comparison to the non-denoised error rate of the samples. As expected, for those samples where both reads were completely overlapping the non-denoised error rates for both mock communities were low. As such, the improvements due to denoising algorithms were found to be minimal. Although, IPED is capable reducing the error rate effectively and robustly throughout all of the read positions, it is more pronounced when both paired reads are not completely overlapping, with the most significant improvement for the second read.

**
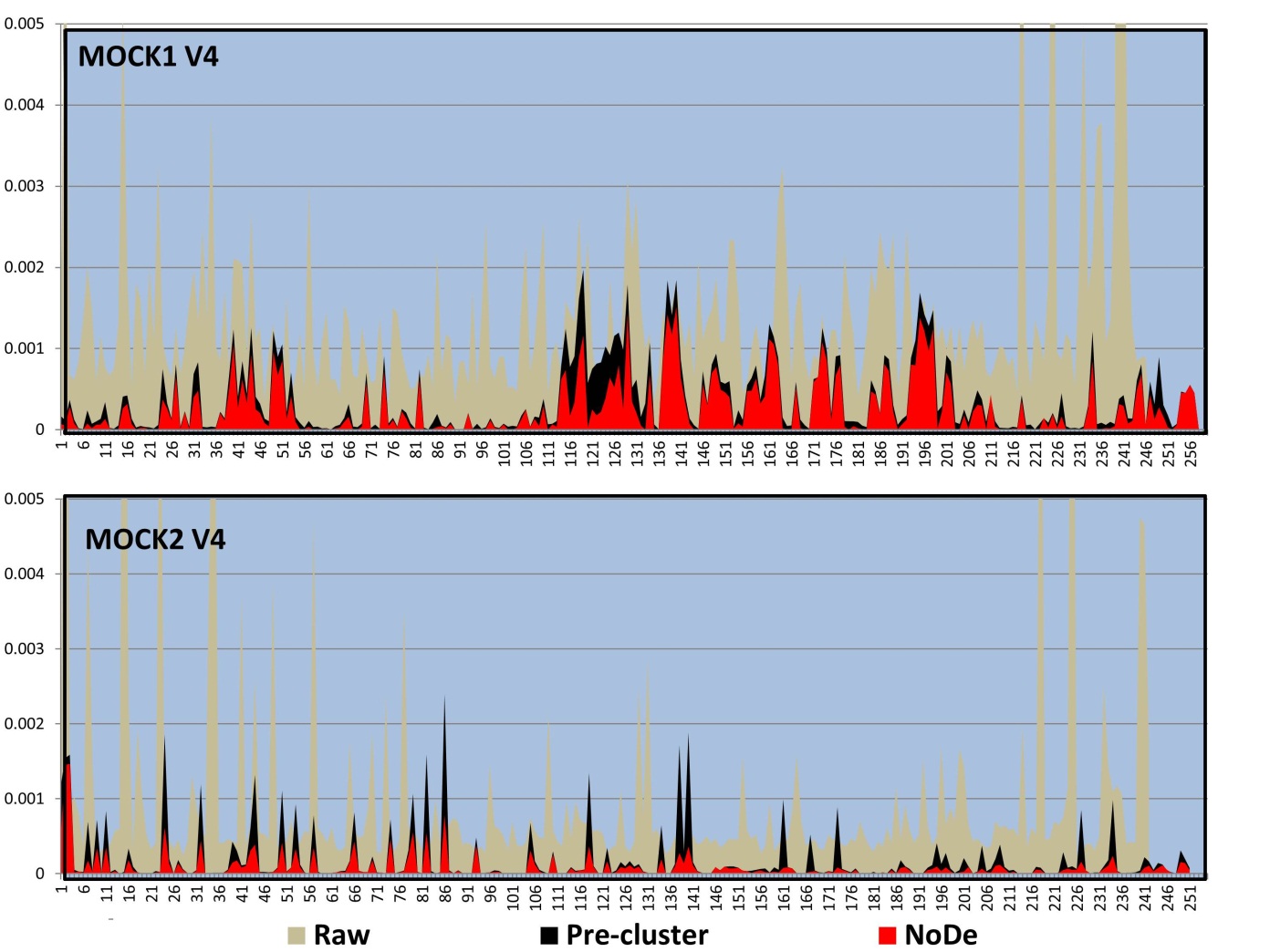
**

Supplementary figure 6 Plot showing the error rate versus the position in the read after being treated with Pre-cluster (black) and IPED (red) with the non-denoised error rate in grey. The overlapping regions are shown the in blue box for V4 in MOCK1 and MOCK2.


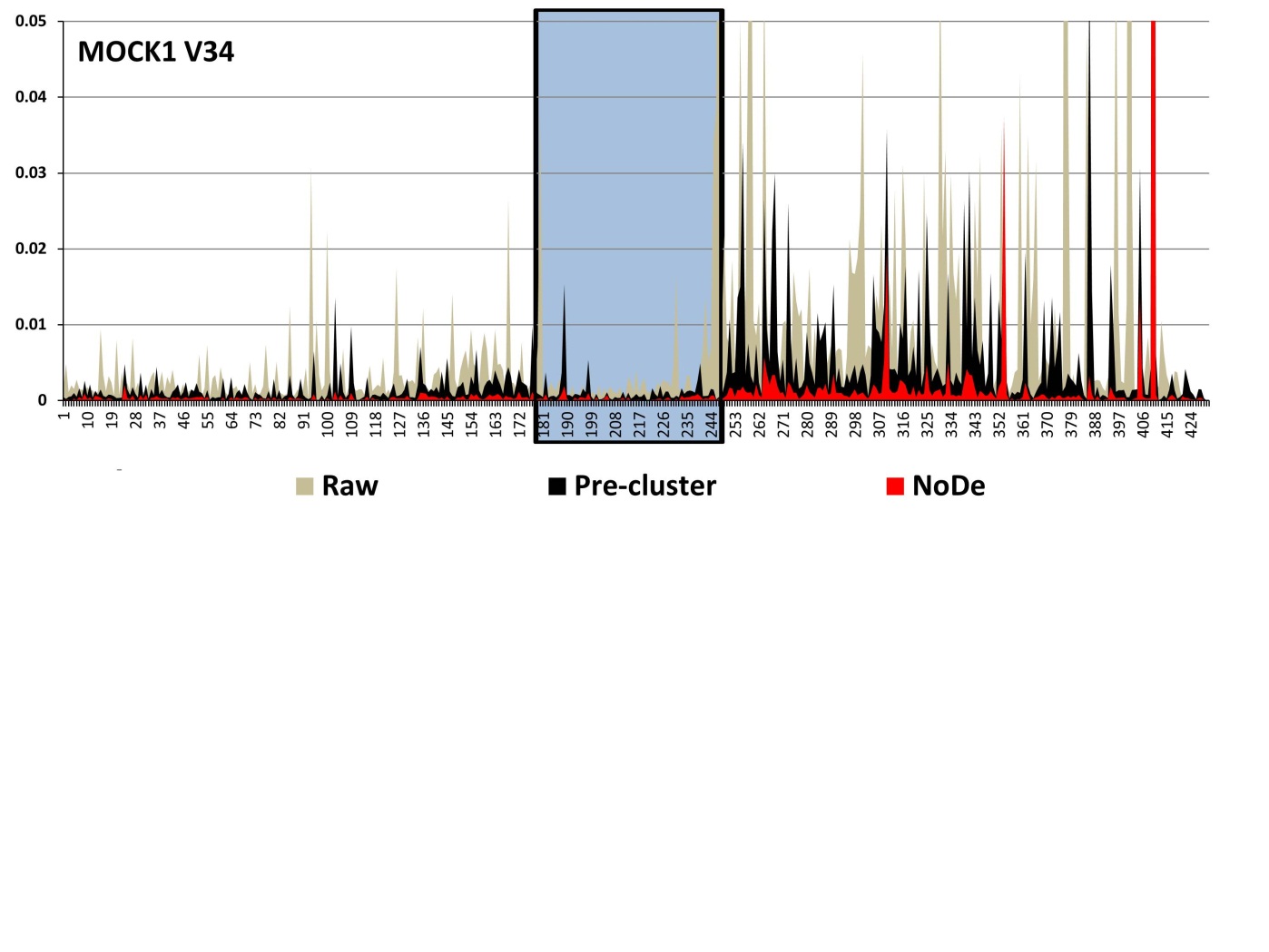
Supplementary figure 7 Plot showing the error rate versus the position in the read after being treated with Pre-cluster (black) and IPED (red) with the non-denoised error rate in grey. The overlapping region is shown in a blue box for V34 in MOCK1.


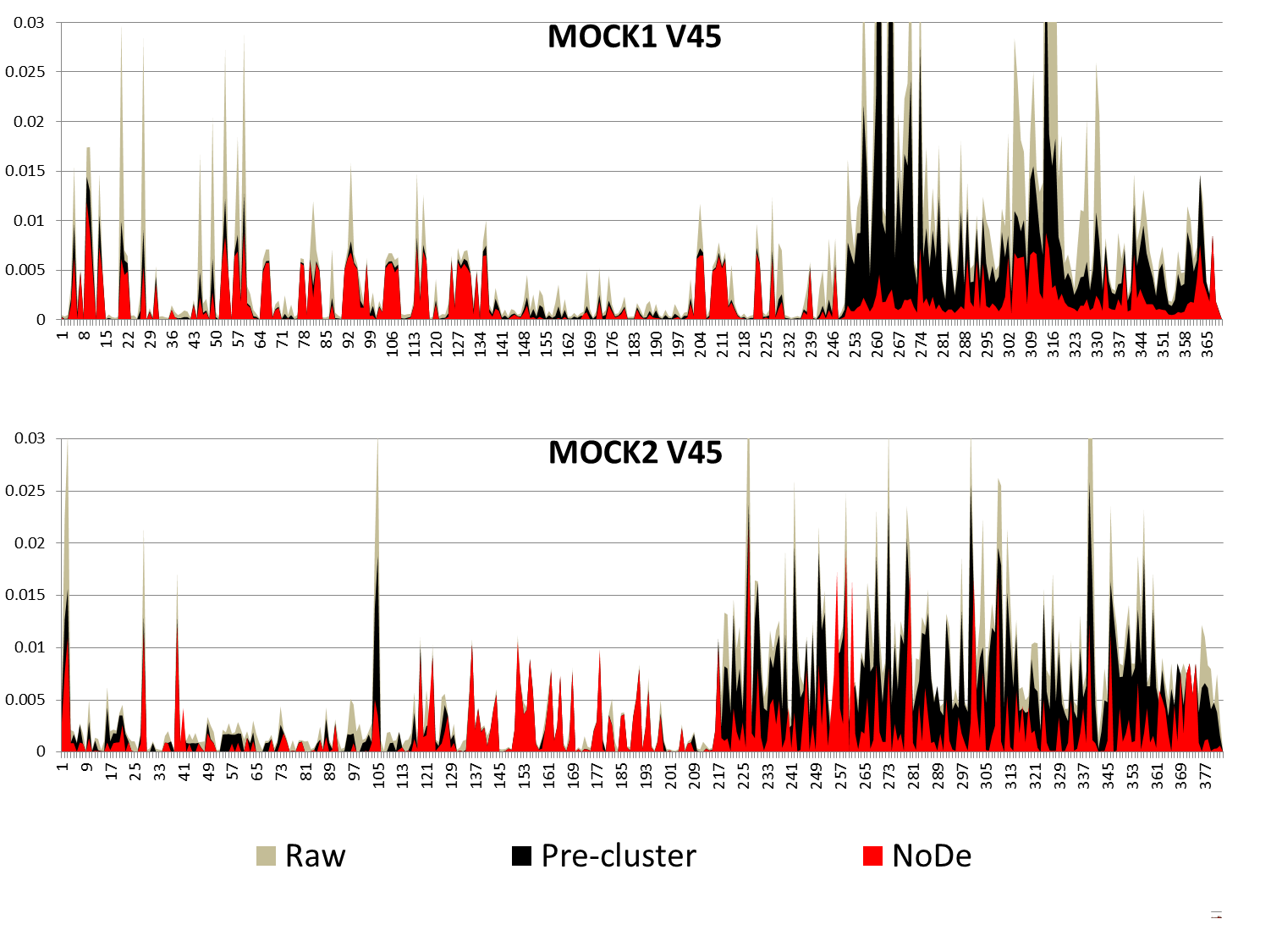


Supplementary figure 8 Plot showing the error rate versus the position in the read after being treated with Pre-cluster (black) and IPED (red) with the non-denoised error rate in grey. The overlapping regions are shown in a blue box for V45 in MOCK1 and MOCK2.

1. **Computational Cost Analysis**

To investigate the extra computational cost related to IPED, the calculation time was registered for all three samples of the MOCK1 dataset covering the V4 region, each sample stratified to 6000 unique reads. When one processor (single Intel Xeon E5-2640 2.50 GHz CPU) was used for each sample (i.e. a total of three processors), IPED required 70 seconds for running all three samples, while Pre-cluster could end the analysis in 14 secs. Taking into account all the different steps required in going from raw Illumina MiSeq 16S rRNA sequencing data to operational taxonomic units (OTUs), this will lead to an increase of 40-50% of the total computational time when IPED is integrated, using either UPARSE or mothur OTU clustering approaches. To evaluate the scalability of both approaches against different datasets sizes, we plotted the computational cost of the individual samples treated with IPED and Pre-cluster. As the unique reads were used to represent each sample, they range from 1,589-131,910 (the total number of reads range from 2,084 to 696,310) IPED showed on average a 5-fold increase of computational costs compared to Pre-cluster.

Supplementary figure 9 Plots showing the computational cost (hours: minutes: seconds) of various pre-processing approaches, where IPED and Pre-cluster were used for denoising, and afterwards followed either by UPARSE (figure A) or mothur (figure B) for OTU calculations, as shown in the methods.


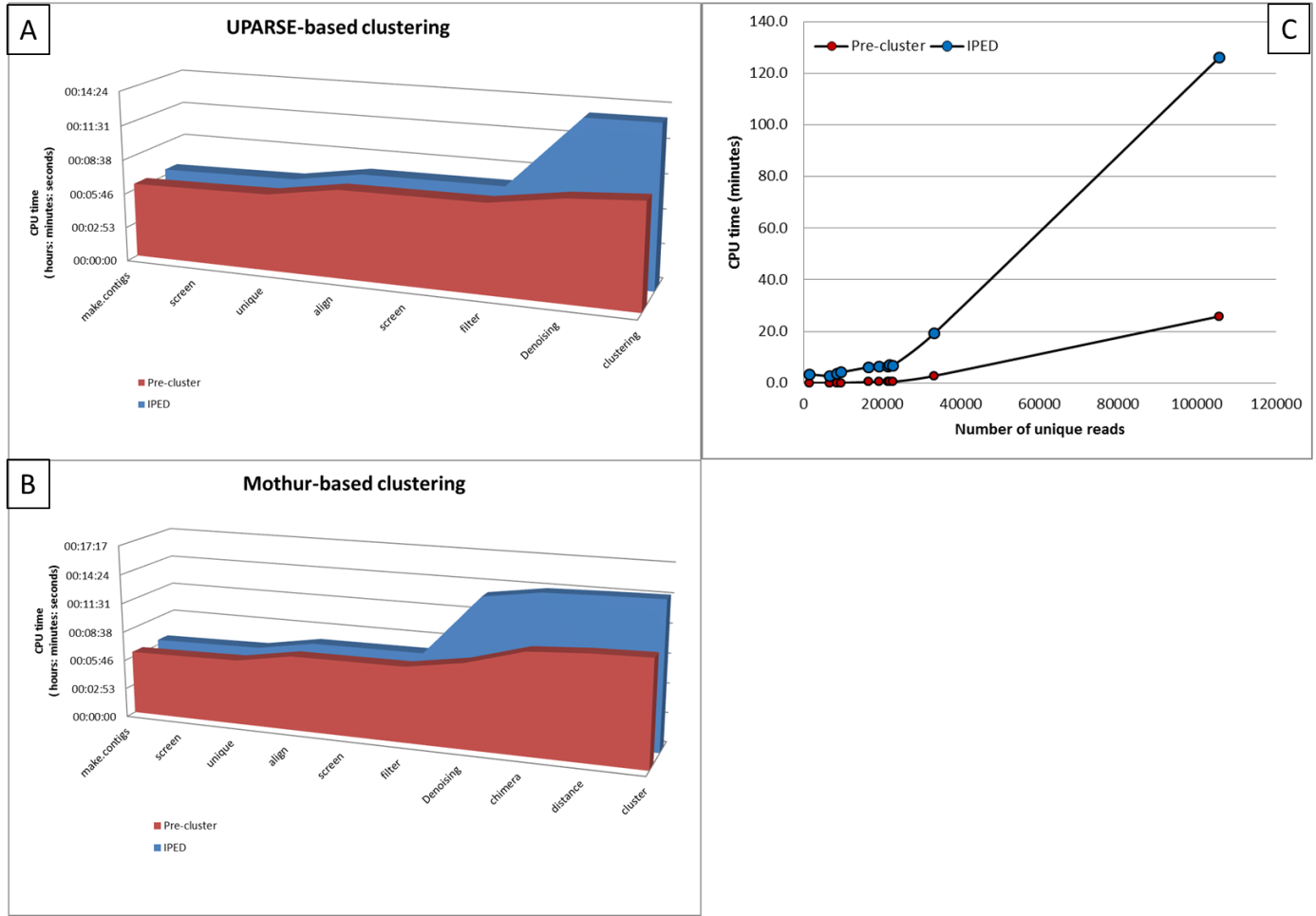


Figure 9 Plot showing the computational cost (hours: minutes: seconds) of various pre-processing approach, where IPED and Pre-cluster were used for denoising and both UPARSE and mothur, were used for OTU calculations, as shown in the methods [A and B respectively]. To evaluate the scalability of both denoising approaches against different datasets sizes, the amount of unique reads from each sample were plotted against the corresponding computational cost (mins), [shown in C].

1. **Denoising Effect on Operational Taxonomic Units (OTUs) Clustering**

## Assessment of OTUs Numbers After Denoising

In principle, the impact of a successful denoising algorithm should be reflected in the number of OTUs. Therefore, we evaluated the number of OTUs for each mock community after applying UPARSE and average neighborhood clustering (implemented in mothur) algorithms respectively. This analysis was done, either with no denoising algorithm (raw), with Pre-cluster, or with IPED, after the removal of chimera (detected by the mothur seq.error command). Ideally, in the complete absence of chimera and sequencing errors, the number of OTUs should be 21 for MOCK1.. We also rarefied the data for all samples to 5,000 or 25,000 sequences per sample, as done in Kozich *et al*. [1].

Supplementary table 12 Evaluation of the number of OTUs produced after treating the data with different denoising algorithms. The left side of the table displays the number of OTUs after using all of the sequences. The right side of the table illustrates the number of OTUs after rarefying the data to 5,000 and 25,000 sequences per sample respectively. The top of the table shows the results when using Nearest Neighbourhood clustering, the bottom of the table displays the results using UPARSE for OTU clustering. Due to computational difficulties we could not calculate OTUs MOCK1 V45.

| **Mothur Clustering Algorithm (cluster)** | | | | | | | | | | | | | | | | |
| --- | --- | --- | --- | --- | --- | --- | --- | --- | --- | --- | --- | --- | --- | --- | --- | --- |
| mock name | ID | **All sequence** | | | | | **5,000 Rarefaction** | | | | | **25,000 Rarefaction** | | | | |
|  |  | **USEARCH** | **USEARCH + UNOISE** | **Mothur** | **Mothur + Pre-cluster** | **Mothur + IPED** | **UPARSE** | **UNOISE** | **Mothur** | **Mothur + Pre-cluster** | **IPED** | **UPARSE** | **UNOISE** | **Mothur** | **Mothur + Pre-cluster** | **Mothur + IPED** |
| **MOCK1** | 130403(**V34)** | 15 | 15 | 58 | 58 | 45 | 15 | 15 | 26 | 29 | 25 | 15 | 15 | 58 | 58 | 45 |
|  | 130417(**V34)** | 15 | 15 | 52 | 52 | 39 | 15 | 15 | 26 | 30 | 25 | 15 | 15 | 52 | 52 | 39 |
|  | 130422(**V34)** | 15 | 15 | 83 | 87 | 61 | 15 | 15 | 32 | 36 | 28 | 15 | 15 | 83 | 87 | 61 |
|  | 130403(**V4)** | 204 | 194 | 165 | 168 | 135 | 25 | 23 | 23 | 23 | 23 | 42 | 38 | 38 | 37 | 34 |
|  | 130417(**V4)** | 168 | 163 | 142 | 140 | 118 | 24 | 23 | 22 | 22 | 22 | 36 | 34 | 34 | 33 | 32 |
|  | 130422(**V4)** | 147 | 145 | 123 | 129 | 109 | 23 | 23 | 22 | 23 | 22 | 36 | 34 | 34 | 34 | 31 |
| **MOCK2** | v4.I.1 | 43 | 48 | 58 | 56 | 55 | 20 | 19 | 20 | 21 | 20 | 25 | 23 | 26 | 26 | 25 |
|  | v4.I.05 | 52 | 52 | 57 | 57 | 52 | 20 | 21 | 20 | 21 | 20 | 26 | 23 | 26 | 27 | 26 |
|  | v4.v5.1 | 59 | 59 | 54 | 50 | 29 | 59 | 59 | 29 | 50 | 29 | 59 | 59 | 54 | 50 | 29 |
|  | v4.v5.I.11 | 669 | 666 | 200 | 186 | 133 | 174 | 173 | 38 | 45 | 38 | 437 | 435 | 128 | 120 | 91 |
| **MOCK3** | M1(**V34)** | 101 | 100 | 85 | 85 | 83 | 49 | 48 | 42 | 43 | 42 | 101 | 100 | 85 | 85 | 83 |
|  | M2(**V34)** | 148 | 148 | 123 | 123 | 113 | 52 | 51 | 42 | 43 | 42 | 130 | 127 | 109 | 112 | 103 |
|  | M3(**V34)** | 74 | 74 | 61 | 60 | 57 | 47 | 46 | 37 | 40 | 37 | 74 | 74 | 61 | 61 | 57 |
| mock name | ID | **All sequence** | | | | | **5,000 Rarefaction** | | | | | **25,000 Rarefaction** | | | | |
|  |  | **USEARCH** | **USEARCH + UNOISE** | **Mothur** | **Mothur + Pre-cluster** | **Mothur + IPED** | **UPARSE** | **UNOISE** | **Mothur + IPED** | **Mothur + Pre-cluster** | **Mothur + IPED** | **UPARSE** | **UNOISE** | **Mothur** | **Mothur + Pre-cluster** | Mothur + IPED |
| **MOCK1** | 130403(**V34)** | 15 | 15 | 51 | 50 | 43 | 15 | 15 | 29 | 28 | 22 | 15 | 15 | 51 | 50 | 43 |
|  | 130417(**V34)** | 15 | 15 | 45 | 42 | 40 | 15 | 15 | 30 | 26 | 23 | 15 | 15 | 45 | 42 | 39 |
|  | 130422(**V34)** | 15 | 15 | 71 | 69 | 55 | 15 | 15 | 40 | 37 | 25 | 15 | 15 | 71 | 69 | 55 |
|  | 130403(**V4)** | 191 | 184 | 158 | 152 | 124 | 25 | 21 | 24 | 23 | 22 | 39 | 37 | 39 | 36 | 34 |
|  | 130417(**V4)** | 157 | 155 | 139 | 136 | 112 | 23 | 24 | 23 | 22 | 22 | 34 | 36 | 36 | 32 | 31 |
|  | 130422(**V4)** | 142 | 135 | 116 | 120 | 102 | 25 | 23 | 23 | 23 | 22 | 37 | 27 | 33 | 34 | 30 |
| **MOCK2** | v4.I.1 | 36 | 57 | 32 | 32 | 29 | 37 | 22 | 20 | 20 | 20 | 44 | 26 | 23 | 23 | 22 |
|  | v4.I.05 | 36 | 53 | 31 | 31 | 26 | 42 | 20 | 23 | 22 | 20 | 51 | 23 | 26 | 26 | 21 |
|  | v4.v5.1 | 23 | 27 | 44 | 43 | 25 | 22 | 21 | 44 | 43 | 25 | 22 | 20 | 44 | 43 | 25 |
|  | v4.v5.I.11 | 33 | 28 | 92 | 92 | 46 | 23 | 21 | 29 | 28 | 23 | 24 | 23 | 60 | 59 | 34 |
| **MOCK3** | M1(**V34)** | 41 | 45 | 42 | 42 | 39 | 23 | 24 | 24 | 22 | 21 | 41 | 45 | 39 | 42 | 39 |
|  | M2(**V34)** | 60 | 59 | 55 | 55 | 47 | 23 | 23 | 23 | 23 | 21 | 59 | 55 | 53 | 50 | 43 |
|  | M3(**V34)** | 31 | 30 | 33 | 32 | 28 | 19 | 23 | 26 | 26 | 20 | 31 | 30 | 33 | 32 | 28 |

## Assessment of OTUs Alpha Diversity using Rarefaction Curves

To have detailed inspection on the effect of the denoising on alpha diversity, we show how the different rarefaction curves of each sample after being treated by IPED and Pre-cluster. To emphasis the differential effect of the clustering algorithms we used both mothur approach (nearest neighborhood) and UPARSE approach (see method section). Due to enormous computational demands we were not able to calculate the distances (hence the OTUs) for V45 in MOCK1. The plots show a consistent lower diversity of IPED treated samples over Pre-cluster treated samples as well as the non-denoised samples. Samples containing reads with a complete overlap between the forward and reverse read (V4 samples) showed the least improvement, while significant improvement is observed for not completely overlapping reads, an observation in line with the error rate improvement (see table 2 in the manuscript).


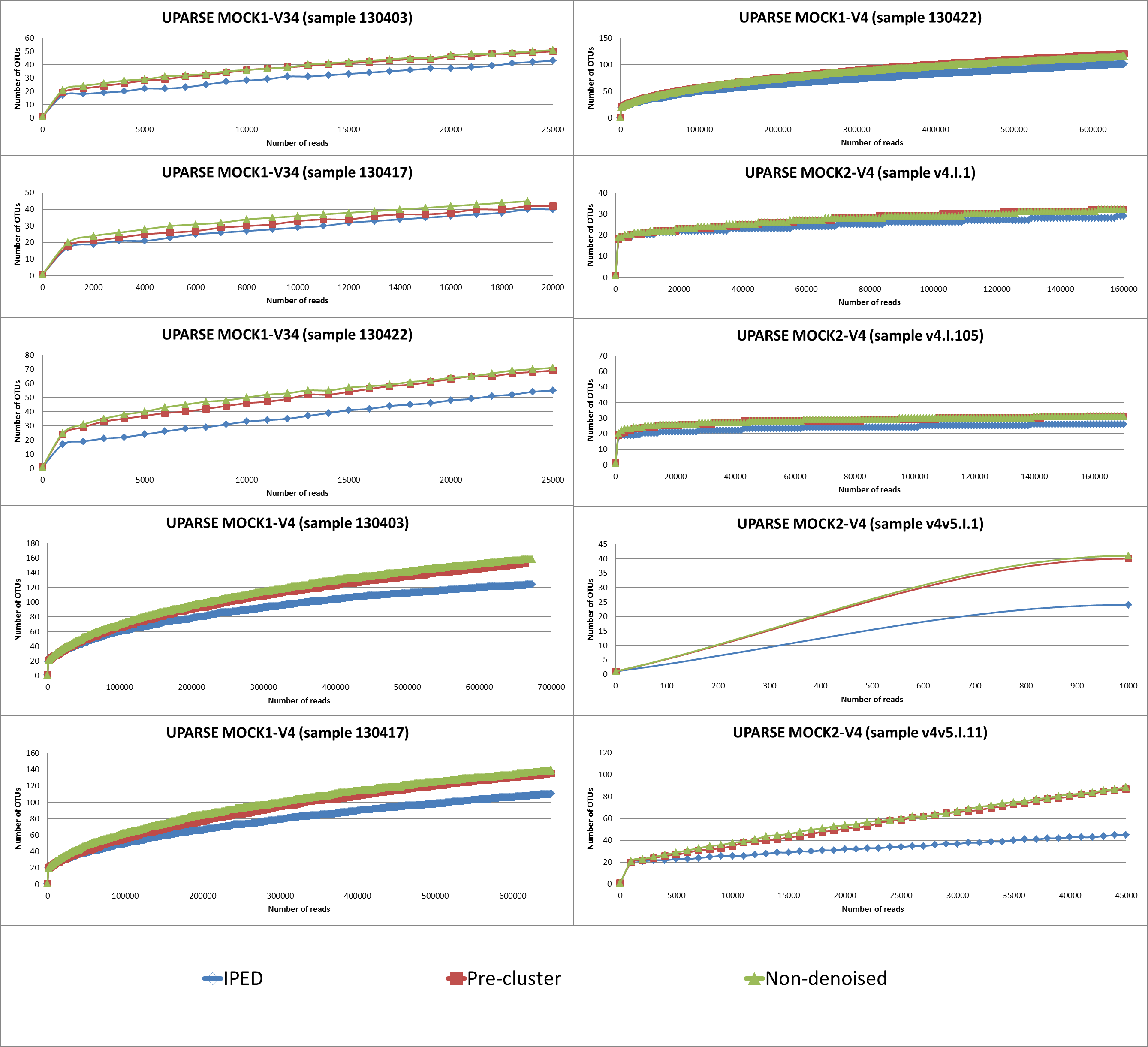


Supplementary figure 10 Rarefaction curves of various samples for the non-denoised reads, together with those being treated by Pre-cluster or IPED, illustrating the positive effect of denoising on reducing the spurious OTUs generated by UPARSE clustering approach.


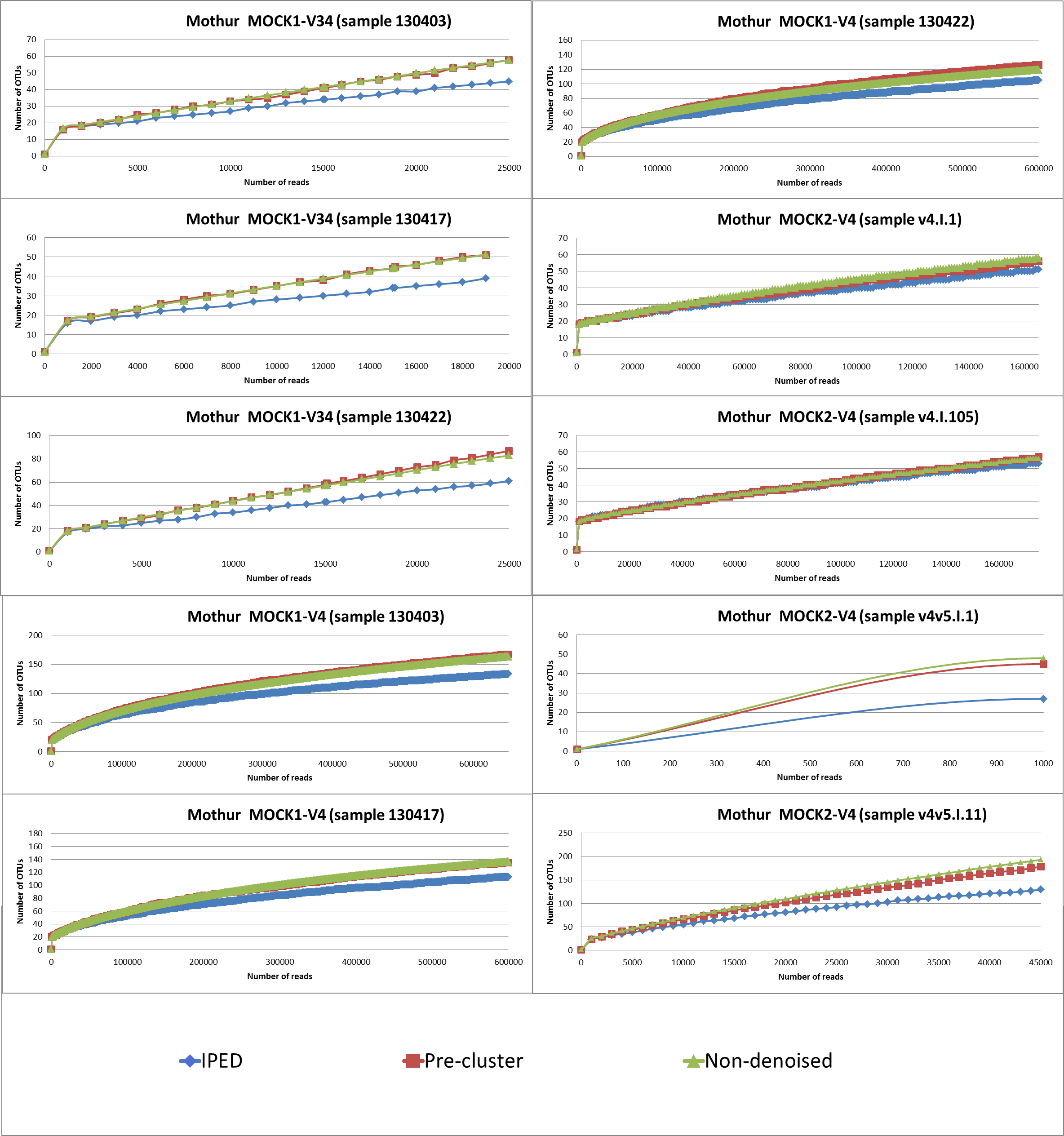


Supplementary figure 11 Rarefaction curves of various samples for the non-denoised reads, together with those being treated by Pre-cluster or IPED, illustrating the positive effect of denoising on reducing the spurious OTUs generated by average neighbourhood clustering approach.

## Assessment of the Impact of the Denoising Algorithms on the Clustering Quality

Both algorithms involve an internal clustering step, where the rare read is removed and its abundance is added to the higher abundance one, whenever it falls within its clustering criteria [as described in the methods]. Therefore, additional tests were proposed to assess the clustering capabilities of both approaches using an objective standard [13]. For reads clustered into other ones after applying both algorithms, we count the true positives (TP), false negatives (FN), true negatives (TN) and false positives (FP). If a pair of reads with a distance below 3% merged into one unique read it is a TP, and if they are not merged, it is a FN. Additionally, if a pair of reads with a distance above 3% merged into one unique read, it is a FP, if they are not merged it is a TN. For this test, we used both mock communities (MOCK1 & MOCK2) with the exception of MOCK1 V45 due to computationally demanding distance calculations. The evaluation was performed using specificity, sensitivity, accuracy and MCC.

Supplementary table 13 Evaluation of clustering obtained via both algorithms Pre-cluster as well as IPED, showing the performance of each algorithm upon tests on MOCK1 & MOCK2 samples (with exception of MOCK1 V45). The evaluation was done using mothur (sens.spec) command.

| Mock | Regions | Approach | ID | sensitivity | specificity | accuracy | mcc |
| --- | --- | --- | --- | --- | --- | --- | --- |
| MOCK1 | V34 | **IPED** | 130403 | 0.806 | 1.000 | 0.982 | 0.888 |
|  |  |  | 130417 | 0.841 | 1.000 | 0.985 | 0.909 |
|  |  |  | 130422 | 0.845 | 1.000 | 0.987 | 0.912 |
|  | V4 |  | 130403 | 0.964 | 1.000 | 0.998 | 0.980 |
|  |  |  | 130417 | 0.961 | 1.000 | 0.997 | 0.974 |
|  |  |  | 130422 | 0.964 | 1.000 | 0.998 | 0.979 |
| MOCK2 | V4 |  | v4.I.1 | 0.888 | 0.999 | 0.992 | 0.934 |
|  |  |  | v4.I.05 | 0.851 | 0.999 | 0.988 | 0.908 |
|  | V45 |  | v4.v5.I.1 | 0.851 | 0.998 | 0.986 | 0.898 |
|  |  |  | v4.v5.I.11 | 0.655 | 1.000 | 0.974 | 0.794 |
| MOCK1 | V34 | **Pre-cluster** | 130403 | 0.317 | 1.000 | 0.936 | 0.544 |
|  |  |  | 130417 | 0.389 | 1.000 | 0.944 | 0.605 |
|  |  |  | 130422 | 0.322 | 1.000 | 0.944 | 0.551 |
|  | V4 |  | 130403 | 0.864 | 1.000 | 0.992 | 0.926 |
|  |  |  | 130417 | 0.868 | 1.000 | 0.992 | 0.928 |
|  |  |  | 130422 | 0.887 | 1.000 | 0.994 | 0.938 |
| MOCK2 | V4 |  | v4.I.1 | 0.784 | 1.000 | 0.985 | 0.878 |
|  |  |  | v4.I.05 | 0.723 | 1.000 | 0.980 | 0.841 |
|  | V45 |  | v4.v5.I.1 | 0.305 | 1.000 | 0.946 | 0.537 |
|  |  |  | v4.v5.I.11 | 0.404 | 1.000 | 0.957 | 0.622 |

Next we performed a qualitative analysis on the OTUs, illustrating the absence of over-clustering (two species represented by one OTU) caused by IPED in comparison to Pre-cluster and UNOISE (with exception of one sample for UNOISE). Under-clustering (same species represented by more than one OTU) is illustrated with over-splitting of the OTUs, showing a more beneficial effect for IPED compared to Pre-cluster or UNOISE in reducing the number of under-clustering incidences. It is important to mention that we forced each read to be classified only into one of the intended species, neglecting any possibility of contamination (as this cannot be properly quantified).

Supplementary table 14 Qualitative analysis to the OTUs produced via UPARSE after being treated with IPED, Pre-cluster, or UNOISE, illustrating the over/under clustering occasions.

|  | MOCK1 | MOCK1 V34-130403 | | | MOCK1 V34-130417 | | | | MOCK1 V34-130422 | | | MOCK1 V4-130403 | | | MOCK1 V4-130417 | | |
| --- | --- | --- | --- | --- | --- | --- | --- | --- | --- | --- | --- | --- | --- | --- | --- | --- | --- |
|  | Ideal Number | IPED | Pre-cluster | UNOISE | IPED | Pre-cluster | UNOISE | | IPED | Pre-cluster | UNOISE | IPED | Pre-cluster | UNOISE | IPED | Pre-cluster | UNOISE |
| Acinetobacter | 1 | 5 | 7 | 1 | 3 | 3 | 1 | | 7 | 8 | 1 | 1 | 2 | 2 | 2 | 2 | 3 |
| Actinomyces | 1 | 1 | 1 | 1 | 1 | 1 | 1 | | 2 | 2 | 1 | 17 | 20 | 23 | 12 | 13 | 18 |
| Bacillus | 1 | 2 | 3 | 1 | 1 | 1 | 1 | | 2 | 3 | 1 | 9 | 11 | 10 | 10 | 13 | 12 |
| Bacteroides | 1 | 3 | 3 | 1 | 6 | 6 | 1 | | 4 | 4 | 1 | 5 | 5 | 6 | 6 | 7 | 6 |
| Clostridium_sensu_stricto | 1 | 0 | 0 | 0 | 0 | 0 | 0 | | 0 | 0 | 0 | 3 | 3 | 3 | 3 | 3 | 3 |
| Deinococcus | 1 | 0 | 0 | 0 | 0 | 0 | 0 | | 0 | 0 | 0 | 5 | 7 | 8 | 2 | 5 | 3 |
| Enterococcus | 1 | 3 | 2 | 1 | 1 | 1 | 1 | | 2 | 2 | 1 | 1 | 1 | 1 | 1 | 1 | 1 |
| Escherichia_Shigella | 1 | 2 | 2 | 1 | 3 | 3 | 1 | | 3 | 3 | 1 | 10 | 12 | 18 | 9 | 10 | 12 |
| Helicobacter | 1 | 0 | 0 | 0 | 0 | 0 | 0 | | 0 | 0 | 0 | 3 | 2 | 4 | 1 | 1 | 2 |
| Lactobacillus | 1 | 7 | 9 | 1 | 9 | 12 | 1 | | 17 | 29 | 1 | 1 | 3 | 3 | 1 | 1 | 1 |
| Listeria | 1 | 2 | 2 | 1 | 2 | 2 | 1 | | 2 | 2 | 1 | 14 | 17 | 22 | 13 | 19 | 20 |
| Neisseria | 1 | 2 | 2 | 1 | 1 | 1 | 1 | | 1 | 1 | 1 | 1 | 1 | 5 | 1 | 1 | 1 |
| Propionibacterium | 1 | 0 | 0 | 0 | 0 | 0 | 0 | | 0 | 0 | 0 | 6 | 6 | 9 | 5 | 7 | 9 |
| Pseudomonas | 1 | 5 | 5 | 1 | 3 | 2 | 1 | | 2 | 2 | 1 | 1 | 1 | 1 | 2 | 2 | 2 |
| Porphyromonas | 1 | 1 | 1 | 1 | 1 | 1 | 1 | | 1 | 1 | 1 | 10 | 12 | 12 | 7 | 9 | 10 |
| Rhodobacter | 1 | 0 | 0 | 0 | 0 | 0 | 0 | | 0 | 0 | 0 | 33 | 45 | 52 | 33 | 37 | 48 |
| Staphylococcus | 1 | 5 | 8 | 1 | 6 | 6 | 1 | | 8 | 7 | 1 | 1 | 1 | 1 | 1 | 2 | 1 |
| Streptococcus | 3 | 5 | 5 | 3 | 3 | 3 | 3 | | 4 | 5 | 3 | 3 | 3 | 4 | 3 | 3 | 3 |
|  | MOCK2 | MOCK2 v4.v5.I.1 | | | MOCK2 v4.v5.I.11 | | | | MOCK2 v4.I.1 | | | MOCK2 v4.I.105 | | | MOCK1 V4-130422 | | |
|  | Ideal Number | IPED | Pre-cluster | UNOISE | IPED | Pre-cluster | | UNOISE | IPED | Pre-cluster | UNOISE | IPED | Pre-cluster | UNOISE | IPED | Pre-cluster | UNOISE |
| Acinetobacter | 1 | 1 | 2 | 1 | 1 | 1 | | 1 | 1 | 1 | 1 | 1 | 1 | 3 | 2 | 2 | 2 |
| Actinomyces | 1 | 1 | 2 | 1 | 2 | 3 | | 1 | 2 | 2 | 2 | 1 | 1 | 1 | 15 | 15 | 16 |
| Bacillus | 1 | 1 | 1 | 1 | 4 | 6 | | 3 | 2 | 3 | 7 | 1 | 2 | 4 | 8 | 10 | 11 |
| Bacteroides | 1 | 2 | 2 | 2 | 2 | 3 | | 1 | 1 | 1 | 1 | 1 | 1 | 2 | 7 | 7 | 7 |
| Clostridium_sensu_stricto | 1 | 1 | 1 | 2 | 1 | 2 | | 1 | 1 | 1 | 1 | 1 | 1 | 1 | 4 | 4 | 4 |
| Deinococcus | 1 | 1 | 1 | 2 | 1 | 4 | | 1 | 1 | 1 | 1 | 1 | 1 | 1 | 1 | 4 | 3 |
| Enterococcus | 1 | 1 | 1 | 1 | 1 | 2 | | 1 | 1 | 2 | 3 | 2 | 2 | 7 | 1 | 1 | 2 |
| Escherichia_Shigella | 1 | 1 | 1 | 1 | 1 | 2 | | 1 | 2 | 2 | 5 | 2 | 2 | 4 | 5 | 6 | 7 |
| Helicobacter | 1 | 1 | 3 | 1 | 2 | 4 | | 1 | 1 | 1 | 1 | 1 | 1 | 2 | 1 | 1 | 1 |
| Lactobacillus | 1 | 2 | 5 | 2 | 1 | 2 | | 1 | 1 | 1 | 3 | 2 | 2 | 4 | 3 | 4 | 5 |
| Listeria | 1 | 1 | 2 | 1 | 5 | 10 | | 2 | 1 | 1 | 6 | 2 | 4 | 4 | 10 | 13 | 16 |
| Neisseria | 1 | 2 | 4 | 2 | 3 | 6 | | 2 | 3 | 3 | 4 | 2 | 3 | 4 | 1 | 1 | 1 |
| Propionibacterium | 1 | 1 | 3 | 1 | 1 | 2 | | 1 | 2 | 2 | 2 | 2 | 2 | 3 | 5 | 5 | 6 |
| Pseudomonas | 1 | 2 | 2 | 2 | 1 | 1 | | 1 | 1 | 1 | 1 | 1 | 1 | 1 | 1 | 1 | 2 |
| Porphyromonas | 0 | 0 | 0 | 0 | 0 | 0 | | 0 | 0 | 0 | 0 | 0 | 0 | 0 | 8 | 11 | 13 |
| Rhodobacter | 1 | 1 | 1 | 1 | 1 | 1 | | 1 | 5 | 5 | 7 | 1 | 1 | 2 | 25 | 30 | 34 |
| Staphylococcus | 1 | 2 | 4 | 2 | 4 | 14 | | 1 | 1 | 2 | 2 | 2 | 3 | 5 | 2 | 2 | 2 |
| Streptococcus | 3 | 4 | 8 | 4 | 15 | 29 | | 8 | 3 | 3 | 10 | 3 | 3 | 5 | 3 | 3 | 3 |

## Assessment of the Influence of Denoising Algorithms on the OTU Clustering Quality

The same test as described in section 6.3 was conducted after the OTU clustering step (UPARSE), where the effect of the denoising algorithms on the quality of the OTU-clustering was evaluated. Based on those results, samples analyzed using IPED were shown to have a higher sensitivity (81% on average) – i.e. less under-clustering – than those treated with Pre-cluster (70% on average), while both algorithms produce similar specificity values (99.1% and 99.4% for IPED and Pre-cluster respectively).

Supplementary table 15 Evaluation of OTUs clusters after running UPARSE on the outputs of both algorithms (Pre-cluster & IPED), showing the effect of each algorithm on UPARSE performance upon tested using MOCK1 & MOCK2 samples. The evaluation was done using the mothur sens.spec command.

| Mock | Regions | Approach | ID | **sensitivity** | **specificity** | **accuracy** | MCC |
| --- | --- | --- | --- | --- | --- | --- | --- |
| **MOCK1** | **V34** | **IPED** | 130403 | 0.924 | 0.974 | 0.971 | 0.802 |
|  |  |  | 130417 | 0.927 | 0.992 | 0.988 | 0.906 |
|  |  |  | 130422 | 0.901 | 0.993 | 0.988 | 0.883 |
|  | **V4** |  | 130403 | 0.989 | 0.995 | 0.995 | 0.873 |
|  |  |  | 130417 | 0.952 | 0.996 | 0.995 | 0.890 |
|  |  |  | 130422 | 0.971 | 0.995 | 0.995 | 0.885 |
|  | **V45** |  | 130403 | 0.449 | 0.998 | 0.995 | 0.447 |
|  |  |  | 130417 | 0.452 | 0.996 | 0.992 | 0.476 |
|  |  |  | 130422 | 0.536 | 0.997 | 0.993 | 0.531 |
| **MOCK2** | **V4** |  | v4.I.1 | 0.893 | 0.994 | 0.989 | 0.874 |
|  |  |  | v4.I.05 | 0.642 | 0.995 | 0.976 | 0.740 |
|  | **V45** |  | v4.v5.I.1 | 0.935 | 0.981 | 0.979 | 0.763 |
|  |  |  | v4.v5.I.11 | 0.935 | 0.981 | 0.979 | 0.764 |
| **MOCK1** | **V34** | **Pre-cluster** | 130403 | 0.916 | 0.998 | 0.990 | 0.939 |
|  |  |  | 130417 | 0.832 | 0.999 | 0.984 | 0.896 |
|  |  |  | 130422 | 0.688 | 0.999 | 0.971 | 0.809 |
|  | **V4** |  | 130403 | 0.974 | 0.996 | 0.995 | 0.929 |
|  |  |  | 130417 | 0.949 | 0.997 | 0.995 | 0.928 |
|  |  |  | 130422 | 0.979 | 0.994 | 0.994 | 0.919 |
|  | **V45** |  | 130403 | 0.096 | 0.999 | 0.945 | 0.281 |
|  |  |  | 130417 | 0.082 | 0.999 | 0.927 | 0.255 |
|  |  |  | 130422 | 0.091 | 0.999 | 0.937 | 0.269 |
| **MOCK2** | **V4** |  | v4.I.1 | 0.957 | 0.994 | 0.992 | 0.932 |
|  |  |  | v4.I.05 | 0.812 | 0.996 | 0.985 | 0.859 |
|  | **V45** |  | v4.v5.I.1 | 0.850 | 0.980 | 0.974 | 0.744 |
|  |  |  | v4.v5.I.11 | 0.783 | 0.984 | 0.974 | 0.741 |

1. **Denoising Effect on a Real-Life Biological Samples**

To illustrate whether the IPED denoising enhancement persists with real-life biological samples, we used a publicly available data set obtained from the mothur website (<http://www.mothur.org/w/images/d/d6/MiSeqSOPData.zip>), which is provided as a tutorial dataset for evaluation of their proposed Standard Operational Procedure (SOP) (explained in detail in [1]). We followed the general pipeline proposed in this SOP three times, however with modification at the level of the applied error correction algorithm: one time exactly following the SOP (using Pre-cluster for denoising), a second time replacing Pre-cluster with IPED, and a third time without applying any denoising step. We noticed a 12% reduction in the number of OTUs using IPED, while in Pre-cluster it was only 5%.

Supplementary table 16 Effect of both denoising algorithms (Pre-cluster and IPED) on the number of Operational Taxonomic Units.

|  | Operational Taxonomic Units  (OTUs) | | | %improvement | |
| --- | --- | --- | --- | --- | --- |
| Group | Non-denoised | Pre-cluster | IPED | Pre-cluster | IPED |
| F3D0 | 180 | 164 | 153 | 9% | 15% |
| F3D1 | 150 | 146 | 137 | 3% | 9% |
| F3D2 | 192 | 178 | 175 | 7% | 9% |
| F3D3 | 128 | 125 | 119 | 2% | 7% |
| F3D5 | 144 | 139 | 132 | 3% | 8% |
| F3D6 | 162 | 156 | 154 | 4% | 5% |
| F3D7 | 132 | 125 | 112 | 5% | 15% |
| F3D8 | 163 | 158 | 146 | 3% | 10% |
| F3D9 | 172 | 163 | 150 | 5% | 13% |
| F3D141 | 161 | 152 | 141 | 6% | 12% |
| F3D142 | 146 | 140 | 130 | 4% | 11% |
| F3D143 | 138 | 131 | 120 | 5% | 13% |
| F3D144 | 168 | 165 | 140 | 2% | 17% |
| F3D145 | 173 | 174 | 150 | -1% | 13% |
| F3D146 | 171 | 160 | 159 | 6% | 7% |
| F3D147 | 225 | 213 | 186 | 5% | 17% |
| F3D148 | 192 | 175 | 160 | 9% | 17% |
| F3D149 | 206 | 191 | 181 | 7% | 12% |
| F3D150 | 179 | 167 | 152 | 7% | 15% |

The effect was further illustrated on the downstream analysis, as shown in the rarefaction curves (Figure 12) and the PCoA biplots (Figure 13), where for the former analysis a lower number of OTUs will be returned for samples analysed with IPED, while the latter one shows a denser clustering of biologically related samples for IPED compared to Pre-cluster or no denoising.


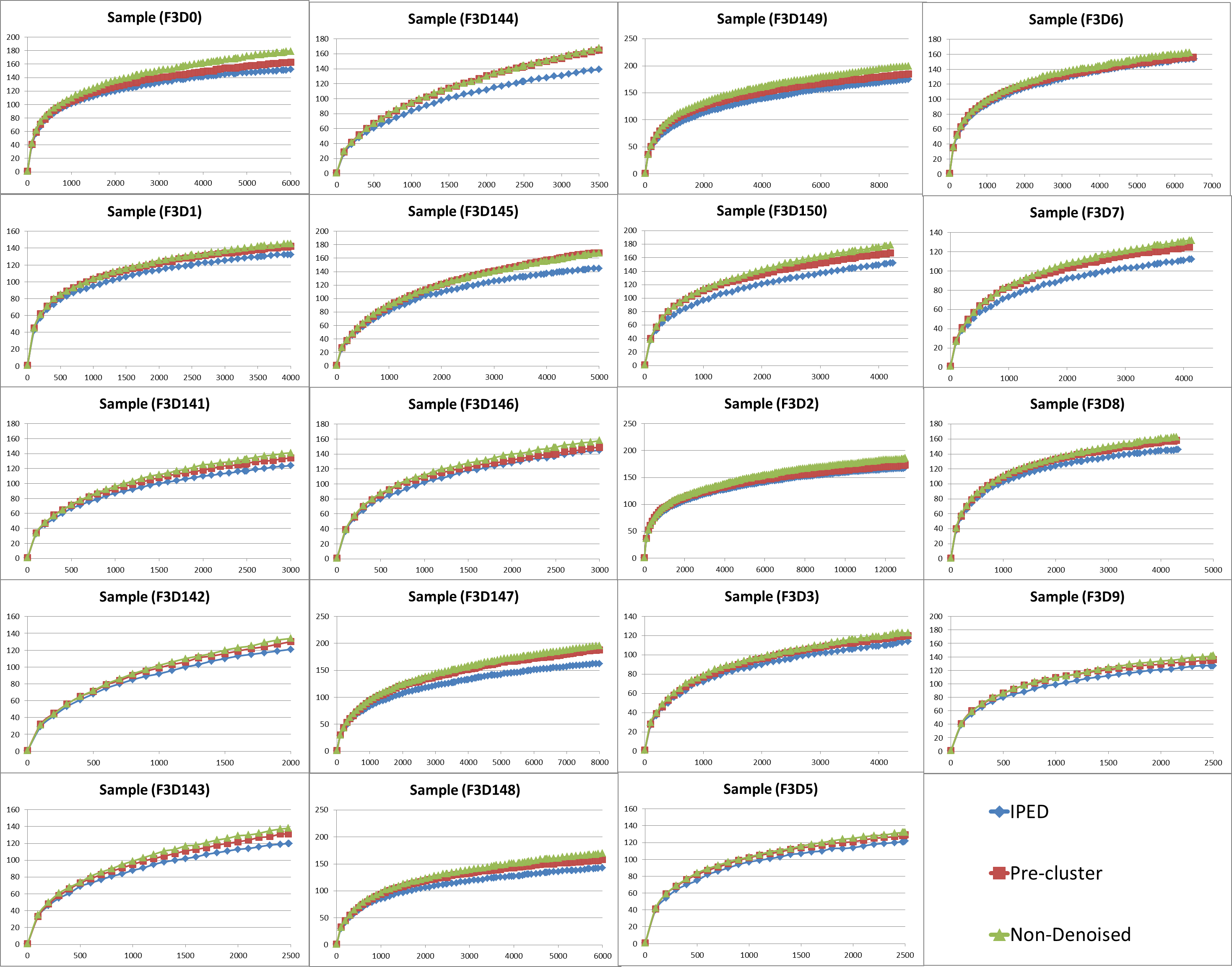


Supplementary figure 12 Illustation of the effect of denoising (IPED, Pre-cluster or no denoising) on the downstream analysis of a real dataset. The number of reads are shown on the "x" axes and the number of OTUs are displayed on the "y" axes..


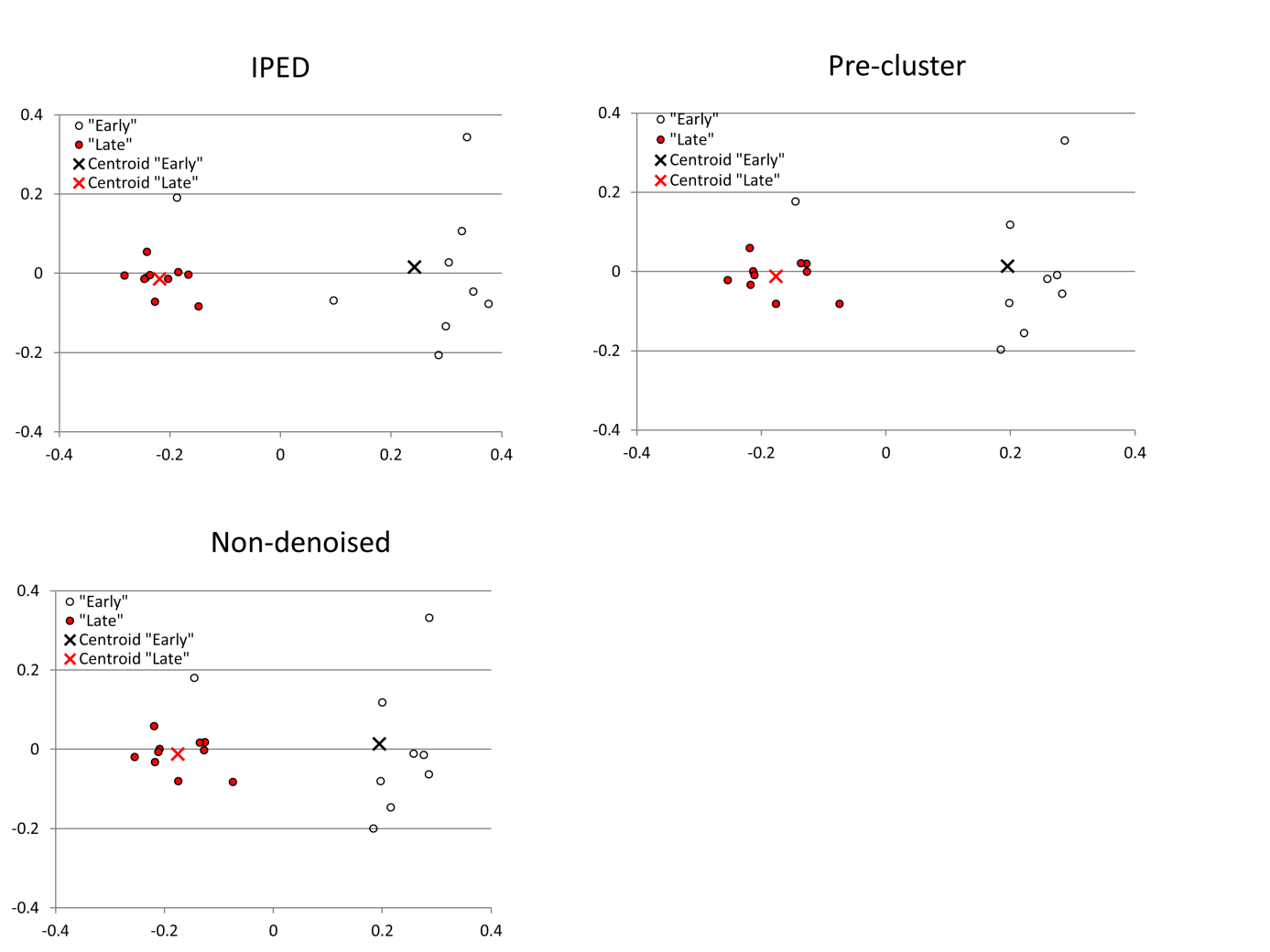


Supplementary figure 13 Illustration of the effect of denoising (IPED, Pre-cluster or no denoising) on the downstream analysis of a real dataset. Based on the PCoA plot, points located close to each other have a similar OTU distribution. As such, a denser (i.e. closer to the centroid) clustering of late versus early stage samples is obtained after applying IPED, compared with Pre-cluster or no denoising.

1. **Effect of presence of chimera and alignment reference database**

To test the effect of the absence of the species from the reference dataset, we removed from the alignment reference the 16S rRNA gene sequences the 12 species composing MOCK3, in addition to any sequence with a distance less than 3% to them (i.e. removing 559 sequences from 14956 sequences in the SILVA dataset). From the results we notice no difference in the error rate of both IPED and Pre-cluster upon using the filtered versus complete reference datasets for alignment.

Moreover, to give a more realistic scenario where a chimera removal algorithm not exposed to the community specific reference database, we applied CATCh (denovo) algorithm, [14], to remove the chimeras for both IPED and Pre-cluster, using MOCK3. As these type of tools are not perfect, they do not remove all of the chimeric reads as well as they do remove some erroneous correct reads (false positive). Hence one can see an increase in the error rate (around 7 folds) basically due to the missed chimeras which would inflate the error rate. In addition, a reduction of the number of OTUs, as a contribution of removal of the false positives. Nonetheless, IPED was able to reduce the error rate with 24% compared to the non-denoised samples, while Pre-cluster and UNOISE reduced the error rate with 11% and 17% respectively. Moreover, the number of OTUs produced after applying IPED was 23% and 6% lower than those produced after applying Pre-cluster and UNOISE respectively (see Table 17). It is important to mention that these results were obtained after the removal of the sequences of the closest species from the alignment reference dataset, as described above.

**Table 17 Illustration of the performance of both IPED, Pre-cluster and UNOISE algorithms upon the removal of chimeras using algorithm exposed to the actual community sequences [Seq.error] and algorithm that was not exposed to it [CATCh].**

| CATCh | Error rate | | | Number of OTUs | | | Total number of Reads | | |
| --- | --- | --- | --- | --- | --- | --- | --- | --- | --- |
|  | M1 | M2 | M3 | M1 | M2 | M3 | M1 | M2 | M3 |
| Non-Denoised | 0.0052 | 0.0056 | 0.0049 | 79 | 119 | 60 | 18213 | 30675 | 11360 |
| IPED | 0.0038 | 0.0044 | 0.0038 | 64 | 85 | 45 | 18534 | 31178 | 11508 |
| Pre-cluster | 0.0047 | 0.0050 | 0.0043 | 96 | 106 | 49 | 16898 | 30482 | 11255 |
| UNOISE | 0.0043 | 0.0048 | 0.0039 | 67 | 92 | 47 | 19955 | 33742 | 12456 |
| Seq.error | Error rate | | | Number of OTUs | | | Total number of Reads | | |
|  | M1 | M2 | M3 | M1 | M2 | M3 | M1 | M2 | M3 |
| Non-Denoised | 0.00149 | 0.0015 | 0.001399 | 85 | 123 | 61 | 18253 | 29553 | 10959 |
| IPED | 0.0004 | 0.0006 | 0.0005 | 84 | 114 | 57 | 18258 | 29553 | 10961 |
| Pre-cluster | 0.0008 | 0.0009 | 0.0008 | 85 | 123 | 60 | 18258 | 28827 | 10707 |
| UNOISE | 0.0006 | 0.0007 | 0.0005 | 100 | 148 | 74 | 19021 | 32004 | 11906 |

1. **References**

1. Kozich JJ, Westcott SL, Baxter NT, Highlander SK, Schloss PD: **Development of a dual-index sequencing strategy and curation pipeline for analyzing amplicon sequence data on the MiSeq Illumina sequencing platform.** *Appl. Environ. Microbiol.* 2013, **79**:5112–20.

2. Nelson MC, Morrison HG, Benjamino J, Grim SL, Graf J: **Analysis, optimization and verification of Illumina-generated 16S rRNA gene amplicon surveys.** *PLoS One* 2014, **9**:e94249.

3. Altschul SF, Gish W, Miller W, Myers EW, Lipman DJ: **Basic local alignment search tool.** *J. Mol. Biol.* 1990, **215**:403–10.

4. Thompson JD, Higgins DG, Gibson TJ: **CLUSTAL W: improving the sensitivity of progressive multiple sequence alignment through sequence weighting, position-specific gap penalties and weight matrix choice.** *Nucleic Acids Res.* 1994, **22**:4673–80.

5. Hall MA: **Correlation-based Feature Selection for Machine Learning**. 1998.

6. He H, Garcia EA: **Learning from imbalanced data**. *IEEE Trans. Knowl. Data Eng.* 2009, **21**:1263–1284.

7. Schloss PD, Westcott SL, Ryabin T, Hall JR, Hartmann M, Hollister EB, Lesniewski RA, Oakley BB, Parks DH, Robinson CJ, Sahl JW, Stres B, Thallinger GG, Van Horn DJ, Weber CF: **Introducing mothur: open-source, platform-independent, community-supported software for describing and comparing microbial communities.** *Appl. Environ. Microbiol.* 2009, **75**:7537–41.

8. Edgar RC: **UPARSE: highly accurate OTU sequences from microbial amplicon reads.** *Nat. Methods* 2013, **10**:996–8.

9. Caporaso JG, Kuczynski J, Stombaugh J, Bittinger K, Bushman FD, Costello EK, Fierer N, Peña AG, Goodrich JK, Gordon JI, Huttley GA, Kelley ST, Knights D, Koenig JE, Ley RE, Lozupone CA, McDonald D, Muegge BD, Pirrung M, Reeder J, Sevinsky JR, Turnbaugh PJ, Walters WA, Widmann J, Yatsunenko T, Zaneveld J, Knight R: **QIIME allows analysis of high-throughput community sequencing data.** *Nat. Methods* 2010, **7**:335–6.

10. Bokulich NA, Subramanian S, Faith JJ, Gevers D, Gordon JI, Knight R, Mills DA, Caporaso JG: **Quality-filtering vastly improves diversity estimates from Illumina amplicon sequencing.** *Nat. Methods* 2013, **10**:57–9.

11. Zhang J, Kobert K, Flouri T, Stamatakis A: **PEAR: a fast and accurate Illumina Paired-End reAd mergeR.** *Bioinformatics* 2014, **30**:614–20.

12. Schirmer M, Ijaz UZ, D’Amore R, Hall N, Sloan WT, Quince C: **Insight into biases and sequencing errors for amplicon sequencing with the Illumina MiSeq platform**. *Nucleic Acids Res.* 2015.

13. Schloss PD, Westcott SL: **Assessing and improving methods used in operational taxonomic unit-based approaches for 16S rRNA gene sequence analysis.** *Appl. Environ. Microbiol.* 2011, **77**:3219–26.

14. Mysara M, Saeys Y, Leys N, Raes J, Monsieurs P: **CATCh, an ensemble classifier for chimera detection in 16S rRNA sequencing studies.** *Appl. Environ. Microbiol.* 2015, **81**:1573–84.
